# Protecting centrosomes from fracturing enables efficient cell navigation

**DOI:** 10.1126/sciadv.adx4047

**Published:** 2025-04-25

**Authors:** Madeleine T. Schmitt, Janina Kroll, Mauricio J. A. Ruiz-Fernandez, Robert Hauschild, Shaunak Ghosh, Petra Kameritsch, Jack Merrin, Johanna Schmid, Kasia Stefanowski, Andreas W. Thomae, Jingyuan Cheng, Gamze Naz Öztan, Peter Konopka, Germán Camargo Ortega, Thomas Penz, Luisa Bach, Dirk Baumjohann, Christoph Bock, Tobias Straub, Felix Meissner, Eva Kiermaier, Jörg Renkawitz

**Affiliations:** ^1^ Biomedical Center, Walter Brendel Center of Experimental Medicine, Institute of Cardiovascular Physiology and Pathophysiology, Klinikum der Universität, Ludwig Maximilians Universität München, Munich, Germany.; ^2^ Institute of Science and Technology Austria, Klosterneuburg, Austria.; ^3^ Life and Medical Sciences (LIMES) Institute, Immune and Tumor Biology, University of Bonn, Bonn, Germany.; ^4^ Bioimaging Facility, Biomedical Center, Faculty of Medicine, Ludwig Maximilians Universität München, Munich, Germany.; ^5^ Institute of Innate Immunity, Department of Systems Immunology and Proteomics, Medical Faculty, University of Bonn, Bonn, Germany.; ^6^ Institute of Stem Cell Research, Helmholtz Center Munich, German Research Center for Environmental Health, Munich, Germany.; ^7^ Physiological Genomics, Biomedical Center, Ludwig-Maximilians University, Munich, Germany.; ^8^ CeMM Research Center for Molecular Medicine of the Austrian Academy of Sciences, Vienna, Austria.; ^9^ Medical Clinic III for Oncology, Hematology, Immuno-Oncology and Rheumatology, University Hospital Bonn, University of Bonn, Bonn, Germany.; ^10^ Medical University of Vienna, Institute of Artificial Intelligence, Center for Medical Data Science, Vienna, Austria.; ^11^ Bioinformatics Unit, Biomedical Center, Faculty of Medicine, Ludwig Maximilians Universität München, Munich, Germany.

## Abstract

The centrosome is a microtubule orchestrator, nucleating and anchoring microtubules that grow radially and exert forces on cargos. At the same time, mechanical stresses from the microenvironment and cellular shape changes compress and bend microtubules. Yet, centrosomes are membraneless organelles, raising the question of how centrosomes withstand mechanical forces. Here, we discover that centrosomes can deform and even fracture. We reveal that centrosomes experience deformations during navigational pathfinding within motile cells. Coherence of the centrosome is maintained by Dyrk3 and cNAP1, preventing fracturing by forces. While cells can compensate for the depletion of centriolar-based centrosomes, the fracturing of centrosomes impedes cellular function by generating coexisting microtubule organizing centers that compete during path navigation and thereby cause cellular entanglement in the microenvironment. Our findings show that cells actively maintain the integrity of the centrosome to withstand mechanical forces. These results suggest that centrosome stability preservation is fundamental, given that almost all cells in multicellular organisms experience forces.

## Teaser

Pathfinding during cellular motility causes centrosome breakage counteracted by Dyrk3 activity.

## INTRODUCTION

Cells inside multicellular organisms experience mechanical forces that originate from intracellular cytoskeletal dynamics and coupling to the extracellular microenvironment, such as to neighboring cells and extracellular matrix (*1*). At the same time, cells have to withstand these forces to prevent damage and preserve functionality, including the maintenance of nuclear integrity when they squeeze through narrow gaps (*2*, *3*). The microtubule cytoskeleton is a major source of intracellular forces, moving cargos like vesicles during interphase and chromosomes during mitosis (*4*). Further, mechanical stresses from the microenvironment and shape changes exert forces on the microtubule cytoskeleton, which typically spans through the entire cell, resulting in microtubule compression or bending (*5*, *6*). In many cells, the centrosome functions as a nucleator and anchor of microtubules (*7*), which suggests that centrosomes evolved an architectural composition primed to withstand forces. Centrosomes are membraneless organelles, composed of a centriolar pair that is connected by non-covalent linker proteins and a surrounding proteinaceous matrix (*4*). While the membraneless property of centrosomes is critical for their timed separation during cell division, linker proteins such as cNAP1 (*CEP250*) connect the centriolar pairs outside of cell division to maintain centrosome cohesion (*8*). However, how the centrosome responds to forces and maintains its integrity under forces remains unknown, raising the general question of how the mechanical integrity of the centrosome is maintained in nondividing cells while experiencing forces.

## RESULTS

### Migration in complex environments deforms the centrosome

**Fig. 1.**
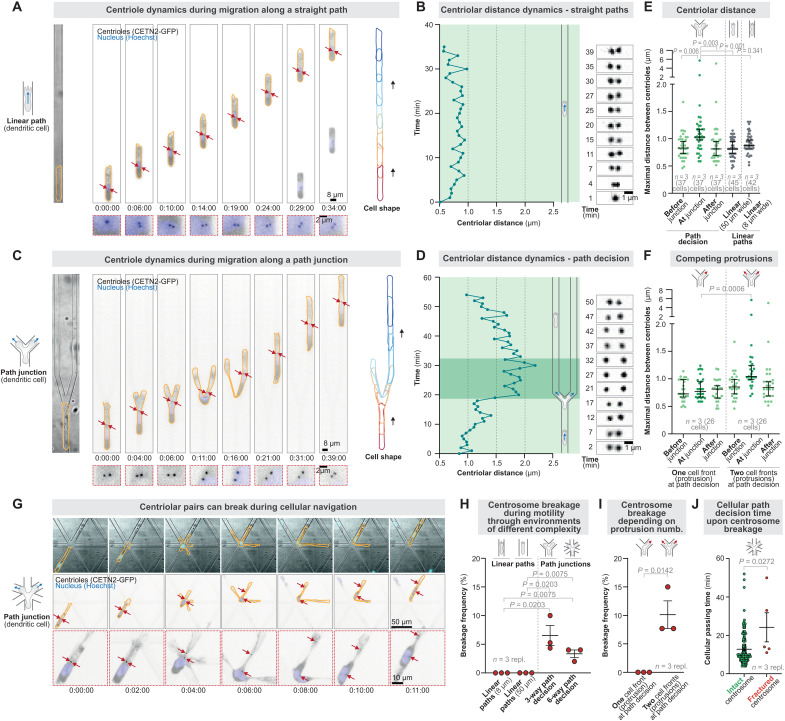
Centrosome deformations and breakage in motile cells. (**A**) Representative CETN2-GFP (centriole pair; black; enlargement in red dashed boxes)–expressing DCs stained with Hoechst (nucleus; blue) migrating along a unidirectional straight path (linear micro-channel). (**B**) Centriolar distance dynamics during migration along a unidirectional straight path; note the stable proximity of the individual centrioles. (**C**) As in (A), but migration through a path junction. Note the two cell fronts exploring the alternative paths. (**D**) Centriolar distance dynamics during a path decision; note the transiently increased distance between the individual centrioles. (**E**) Quantification of centriolar distances before, during, and after migration through path junctions, as well as during migration along narrow and wide linear paths. (**F**) Quantification of centriolar distances of cells that pass the junction either with two simultaneous explorative protrusions or DCs that immediately decide for one path alternative with one protrusion. (**G**) Representative CETN2-GFP (centriole pair; black; enlargement in red dashed boxes)–expressing DCs stained with Hoechst (nucleus; blue) migrating along a six-way path junction. Note the far-distant separation of the two centrioles upon cellular pathfinding. (**H**) Frequency of centrosome breakage during DC migration in environments of different complexity [*n* = 3 replicates (repl.): linear narrow paths, 42 cells; linear wide paths, 45 cells; three-way junctions, 72 cells; six-way junction, 150 cells]. (**I**) Frequency of centrosome breakage during DC migration along a three-way path junction of cells that pass the junction either with two simultaneous explorative protrusions or DCs that immediately decide for one path alternative with one protrusion (*n* = 3 repl.: 1 protrusion, 26 cells; 2 protrusions, 46 cells). (**J**) Path decision time of DCs migrating along a three-way path junction with an intact or fractured centrosome (*n* = 3 repl.: intact centrosome, 145 cells; fractured centrosome, 5 cells). Time is indicated as hours:minutes:seconds.

As a model for cells that experience forces (*9*), we live-imaged motile mouse dendritic cells (DCs) that are terminally differentiated (*10*, *11*) and nucleate microtubules only from the centrosome (*12*). To visualize the centrosome, we used DCs expressing centrin-2 (CETN2)–green fluorescent protein (GFP) (*13*) as a marker for the pair of two centrioles within one centrosome. The two centrioles remained in close proximity of 0.5 to 1 μm with minor distance fluctuations when cells migrated persistently along unidirectional paths along a chemotactic gradient (Fig. 1, A and B, and movie S1), in accordance with stable centriolar distances during centrosome cohesion in interphase cells (*14*). Even during minor directional changes along wider straight paths, the centriole pair moved in a coordinated manner (fig. S1, A and B). Yet, to our surprise, when the cells encountered path junctions, the distances between the pair of centrioles considerably increased, indicating stretching deformations (Fig. 1, C and D, and movie S1). Centrosome stretching was transient, as the initial centriolar distance was restored after productive path decisions (Fig. 1E and movie S1). These results suggested that centrosomes are subject to deformations, likely by forces experienced during migratory pathfinding, in line with historical observations of transient centriole separation during the adhesive spreading of neutrophils (*15*).

Migrating DCs extend simultaneous protrusions into alternative paths, a characteristic feature of motile cells exploring their microenvironment (*16*–*18*). Thus, we compared centrosome deformations in DCs that either explored their path with two competing protrusions or immediately moved into one path with a single protrusion. DCs with competing protrusions deformed their centrosome transiently as the protrusions explored the alternative paths (Fig. 1, C, D, and F). Yet, cells that migrated with a single protrusion did not stretch the centrosome (Fig. 1F and fig. S1, C and D). To confirm centrosomal deformations, we generated DCs expressing the pericentriolar material (PCM) marker Pericentrin-dTomato and observed elongated PCM shapes at path junctions (fig. S9, D and E). These findings show that forces from competing protrusions deform the centrosome. Centrosome stretching deformations rarely resulted in centrosome breakage, but, if so, it only occurred during path exploration with multiple protrusions (Fig. 1, G to I). Given that centrosome breakage caused delays in pathfinding (Fig. 1J), these findings suggested that centrosomes are equipped with mechanisms to withstand forces actively.

### Dyrk3 protects from centrosome fracturing

**Fig. 2.**
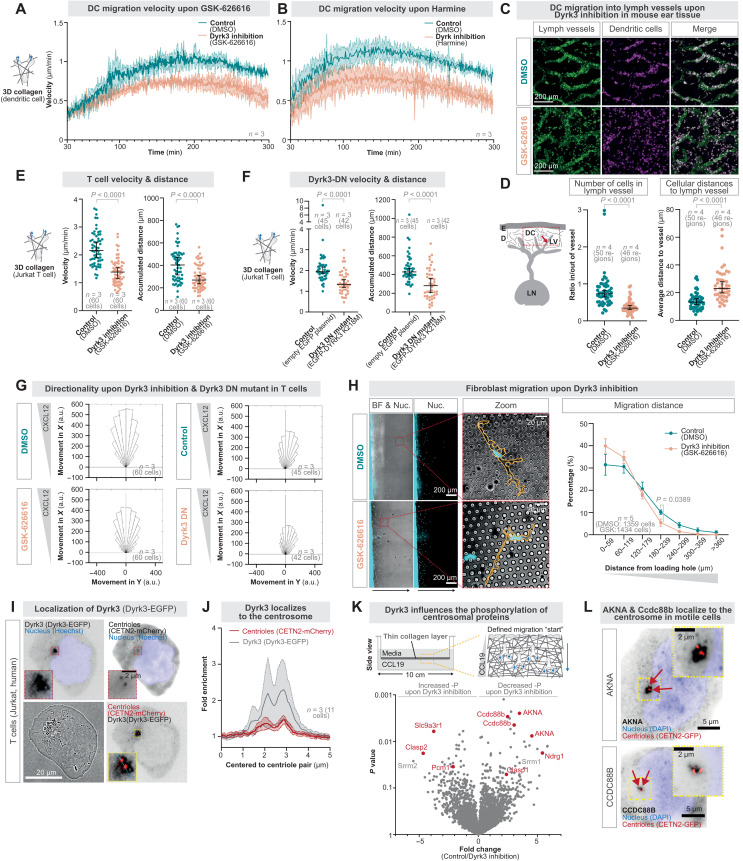
Cell motility requires functional Dyrk3. (**A**) DC migration in three-dimensional (3D) collagen matrices (1.7 mg/ml) along a CCL19 chemokine gradient in the presence of 5 μM GSK-626616 or DMSO (control). (**B**) As in (A), but with 50 μM harmine. (**C**) Representative immunofluorescence staining of in situ migration of DCs (anti-MHCII; magenta) into lymphatic vessels (anti-Lyve1; green) on a mouse ear sheet in the presence of 5 μM GSK-626616 or DMSO (control). (**D**) Quantification of (C), comparing the number of DCs inside to the outside of lymphatic vessels, as well as measuring the closest distance between cells and lymphatic vessels. (**E**) Velocity and migrated distance of Jurkat T cells migrating in 3D collagen matrices (1.3 mg/ml) along a CXCL12 chemokine gradient in the presence of 5 μM GSK-626616 or DMSO (control). (**F**) As in (E), but comparing cells that express a dominant-negative (DN) enhanced green fluorescent protein (EGFP)–Dyrk3 K218 mutant or the corresponding empty EGFP plasmid. (**G**) Directionality along a chemotactic gradient (CXCL12) of Jurkat T cells upon rendering Dyrk3 nonfunctional. a.u., arbitrary units. (**H**) Migrated distance of 3T3 fibroblasts stained with Hoechst (nucleus; cyan) migrating in 3D micropillars in the presence of 5 μM GSK-626616 or DMSO (control). (**I**) Localization of EGFP-tagged WT Dyrk3 and CETN2-mCherry in Jurkat T cells showing a centrosomal localization around the intact pair of centrioles. (**J**) Quantification of (I) by measuring the fluorescent intensity along a 5-μm line [blue dotted line in (I)] centered to the centriole pair. (**K**) Phosphoproteomics of migrating DCs in collagen matrices (1.7 mg/ml) along a CCL19 chemokine gradient in the presence of 5 μM GSK-626616 or DMSO (control). Centrosomal proteins are highlighted in red. (**L**) Immunofluorescence staining of a representative CETN2-GFP (centriole pair; red) expressing Jurkat T cell stained with DAPI (nucleus; blue) and with Akna or CCDC88B (black), respectively.

To screen for mechanisms that maintain centrosome integrity during mechanical deformations, we established transcriptomics of migrating DCs in collagen networks of different complexity (fig. S2A), based on the rationale that motile cells sample more complex environments with more protrusions. Cluster analysis of differentially regulated genes revealed transcriptional adaptation to migration in more complex matrices (fig. S2B), including genes up-regulated in increasing collagen complexity (fig. S2C). Given that the centrosome has features of membraneless biomolecular condensates (*19*–*21*), we were particularly interested in observing the up-regulation of Dyrk3 (fig. S2D), a protein kinase shown to function as a regulator of membraneless organelles (*22*), including its activity at the centrosome for cell cycle progression into mitosis (*23*). To test the role of Dyrk3 for locomotion of nondividing interphase cells, we investigated DCs in the presence of GSK-626616, a well-described small-compound inhibitor of Dyrk3 (*22*, *23*), while migrating through collagen matrices composed of heterogeneously sized pores comparable to tissues, ranging from 1 to 5 μm smaller than the cellular diameter (*24*, *25*). DCs showed reduced velocities during navigation through these matrices (Fig. 2A, fig. S2E, and movie S2), which we also observed in the presence of harmine (Fig. 2B and fig. S2F), an additional small-compound inhibitor of Dyrk-family proteins. Similarly, DC migration toward lymphatic vessels in mouse ear explants and intravasation into the lymphatic vessels was reduced (Fig. 2, C and D). To test generality, we used human Jurkat T cells as another model for fast migration and observed decreased velocities and accumulated distances while maintaining comparable directionality toward a chemokine source in the presence of GSK-626616 (Fig. 2, E to G; fig. S2G; and movie S2). To confirm these findings on a genetic level, we expressed a dominant-negative kinase-dead point mutant of Dyrk3 [enhanced green fluorescent protein (EGFP)–DYRK3 K218M] (*23*) in Jurkat T cells and again observed reduced migration velocities and distances (Fig. 2, F and G, and movie S2). To test whether Dyrk3 plays a role in motility beyond immune cells, we loaded fibroblasts, a model for slow mesenchymal migration, into micropillar forests that mimic complex matrices but offer defined microenvironments. While control cells moved substantially into the microenvironmental mazes, Dyrk3-inhibited and EGFP-DYRK3 K218M expressing cells migrated less (Fig. 2H and fig. S2H). Overall, these findings show that functional Dyrk3 is required for efficient cell migration.

As we observed that Dyrk3 primarily localizes to the centrosome of motile cells (Fig. 2, I and J), we established phosphoproteomics of migrating cells to determine whether Dyrk3 regulates centrosomal proteins. We developed a large-scale chamber where 10 million DCs migrated toward a chemokine inside a collagen matrix (Fig. 2K). Upon phosphopeptide isolation and mass spectrometry, we identified known proteins regulated by Dyrk3, including SRRM1 and SRRM2 (*22*), centrosomal proteins such as PCM1 (*26*) and CLASP1 (*27*), and, more recently, identified centrosomal proteins like AKNA (*28*) and Ndrg1 (*29*), as well as proteins with annotated centrosomal localization like SLC9A3R1 (*30*) and Ccdc88b (*31*) (Fig. 2K). Using immunofluorescence stainings for AKNA and Cdcc88b, we confirmed their localization to the centrosome of motile cells (Fig. 2L). Given the role of Dyrk3 as a biomolecular condensate dissolvase, we tested whether Dyrk3 regulates the physical properties of the centrosome by measuring the diffusion properties of core centrosomal proteins by fluorescence recovery after photobleaching (FRAP). We selected CEP120 as a centrosomal protein with well-known fast diffusion properties (*32*) and observed slower diffusion rates upon rendering Dyrk3 nonfunctional (fig. S3, A and E), indicating altered centrosomal material properties. Similarly, Dyrk3 itself showed quick diffusion properties that were decreased by its inhibition, while the less diffusive proteins AKNA and Pericentrin were not affected (fig. S3, B to E).

**Fig. 3.**
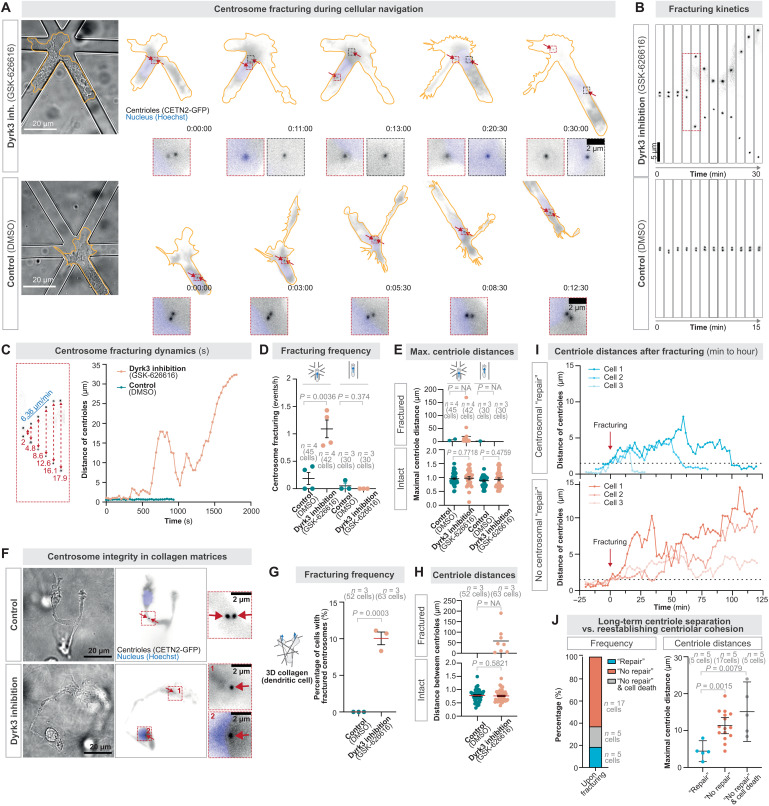
The centrosome fractures during cellular pathfinding in the absence of Dyrk3 activity. (**A**) Dynamics of the centriole pair (CETN2-GFP; black) during DC migration along a six-way path junction in the presence of 5 μM GSK-626616 or DMSO (control). Note the far-distant separation of the two centrioles in the presence of 5 μM GSK-626616. The nucleus is stained with Hoechst (blue). Time is indicated as hours:minutes:seconds. (**B**) As in (A), showing the detailed kinetics of the centriole pair. (**C**) As in (A), showing the detailed velocity of the separation of the centriole pair. (**D**) Frequency of centrosome breakage (defined as distance > 1.5 μm between the centrioles) and (**E**) maximal distance of individual centrioles during DC migration along path junctions (six-way) or unidirectional paths. NA, not applicable; statistical testing is not applicable due to low numbers of fracturing events under control conditions. (**F**) Representative DC during migration in a deformable collagen matrix in the presence of 5 μM GSK-626616 or DMSO (control). (**G**) Percentage of cells with broken centrosomes and (**H**) maximal distance of individual centrioles during DC migration in 3D collagen matrices 2 hours after the start of migration. NA, not applicable; statistical testing is not applicable due to low numbers of fracturing events under control conditions. (**I**) Long-term centriole dynamics during Dyrk3-inhibited (5 μM GSK-626616) DC migration through bead obstacles underneath an agarose layer after centrosome fracturing showing representative examples of “repairing” and “non-repairing” cells. (**J**) As in (I), quantifying the frequency of long-term consequences and corresponding maximal distance of individual centrioles during DC migration upon centrosome fracturing.

**Fig. 4.**
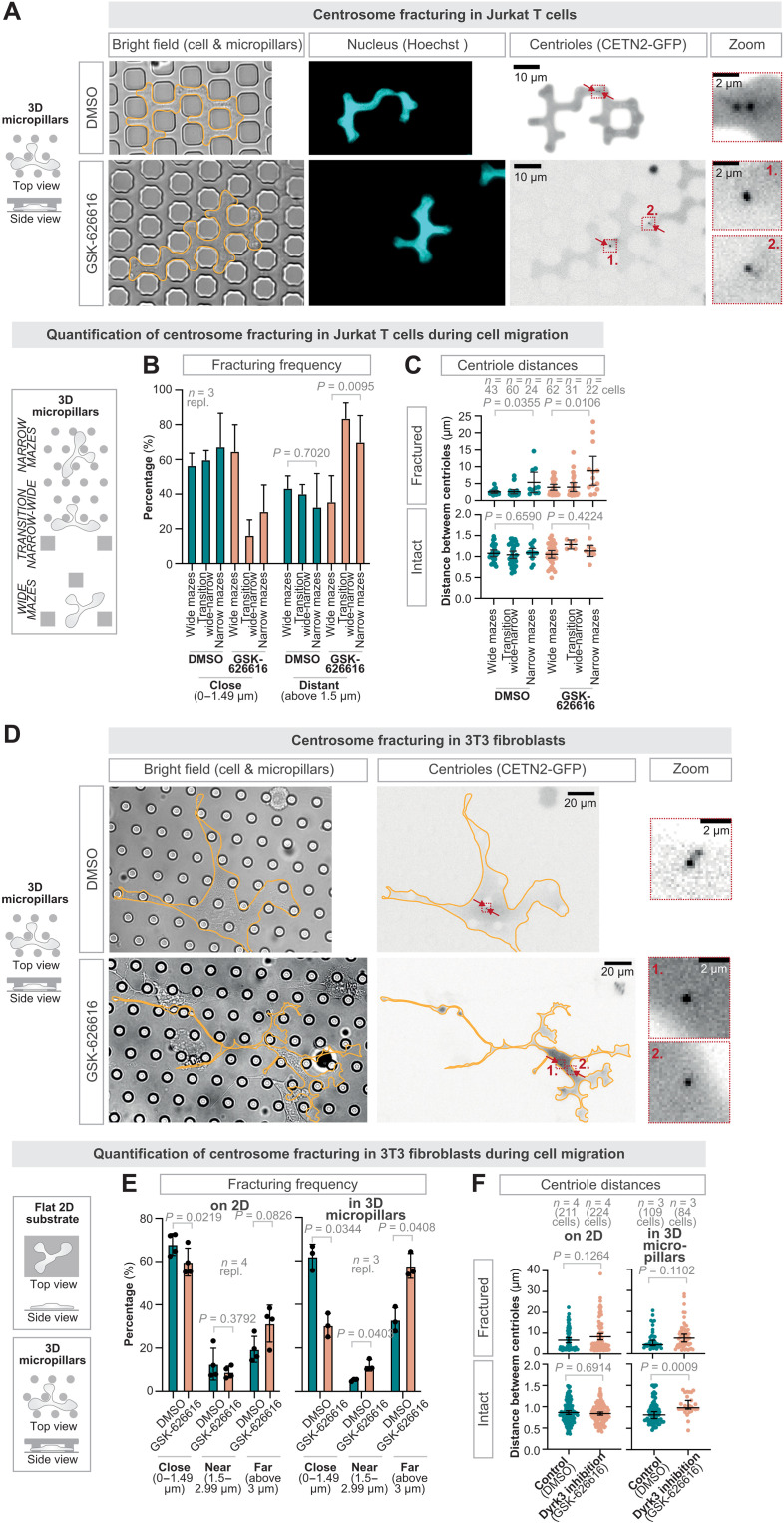
Centrosome fracturing during Jurkat T cell and 3T3 fibroblast migration. (**A**) Representative CETN2-GFP (centriole pair; black; enlargement in red dashed boxes) expressing Jurkat T cells migrating along micropillars in the presence of 5 μM GSK-626616 or DMSO (control). (**B**) Frequency of centrosome breakage classifying the short and long distantly separated individual centrioles, and (**C**) maximal distance of individual centrioles during Jurkat T cell migration in 3D micropillars with narrow and wide mazes in the presence of 5 μM GSK-626616 or DMSO (control) [*n* = 3 repl.; DMSO: 43 cells (wide mazes), 60 cells (transition wide-narrow), and 24 cells (narrow mazes); GSK-626616: 62 cells (wide mazes), 31 cells (transition wide-narrow), and 22 cells (narrow mazes)]. (**D**) Representative CETN2-GFP (centriole pair; black; enlargement in red dashed boxes) expressing 3T3 fibroblasts migrating along micropillars in the presence of 5 μM GSK-626616 or DMSO (control). (**E**) Frequency of centrosome breakage classifying the short and long distantly separated individual centrioles, and (**F**) maximal distance of individual centrioles in 3T3 fibroblasts on 2D and during migration in 3D micropillars with narrow and wide mazes in the presence of 5 μM GSK-626616 or DMSO (control) (*n* = 4 repl.; DMSO: 211 cells on 2D and 109 cells in 3D micropillars; GSK-626616: 224 cells on 2D and 84 cells in 3D micropillars). All data derive from at least three independent biological replicates.

Considering this role of Dyrk3 in regulating centrosomal properties, we tested whether Dyrk3 regulates centrosome integrity. We imaged CETN2-GFP–expressing DCs during navigation through path junctions. Before the path junction, the centrioles located in proximity and moved synchronously (Fig. 3, A and B), indicating centrosome cohesion in intact centrosomes. Yet, once the cells encountered multiple paths, the pair of centrioles frequently broke into far distantly located and individually moving centrioles when Dyrk3 was nonfunctional (Fig. 3, A, D, and E; and movie S3). Centrosome breakage was sudden and fast, with separation velocities in the range of micrometers per minute (Fig. 3, B and C), showing a fracture-like behavior of the centrosome and indicating strong opposing forces acting onto the centrosome. Centrosome fracturing only occurred at path junctions, but not during migration along straight paths (Fig. 3, D and E; fig. S4A; and movie S3). To confirm these results in deformable matrices, we analyzed centrosome integrity during migration in collagen. In the presence of GSK-626616, DCs showed broken pairs of centrioles, resulting in individual centrioles that can be located in different subcellular regions (Fig. 3, F to H). To observe the behavior of broken centriole pairs over time, we established an under-agarose migration assay with bead obstacles to follow cells during pathfinding over hundreds of micrometers (fig. S5, A to E). This revealed that centrioles were able to reestablish cohesion, typically when they only separated for short distances (Fig. 3, I and J). In most cases, however, centrosome fracturing caused long-term splitting of the centriolar pair (Fig. 3, I and J). These findings establish that centrosomes are prone to breakage when Dyrk3 is nonactive and when DCs navigate their paths. To test whether Dyrk3 is generally required to maintain mechanical stability of the centrosome during cell motility, we loaded amoeboid migrating T cells as well as mesenchymal migrating fibroblasts into mazes upon their transfection with CETN2-GFP. While nonactive Dyrk3 did not cause major effects on centrosome integrity when both cell types moved on two-dimensional (2D) substrates, migration within pathfinding mazes led to centrosome fracturing (Fig. 4, A to F). Overall, these data identify that centrosomes are prone to mechanical breakage in the absence of Dyrk3 activity.

**Fig. 5.**
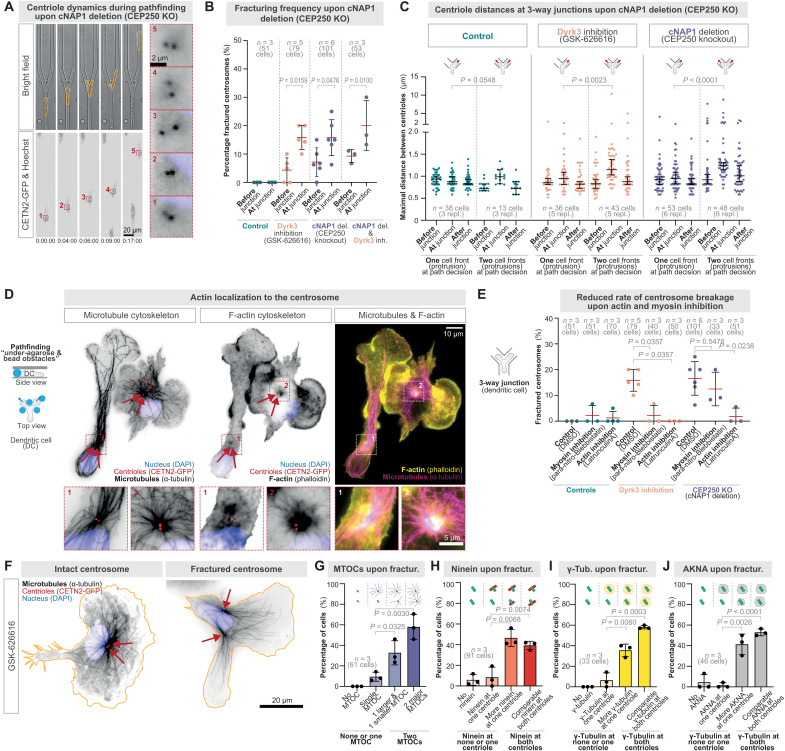
Centrosome fracturing generates two functional MTOCs and is mediated by forces from the actin cytoskeleton. (**A**) Representative CETN2-GFP (centriole pair; black) expressing cNAP1-deficient DC stained with Hoechst (nucleus; blue) migrating along a three-way path junction exploring the alternative paths with one or two cell protrusions. (**B**) Frequency of centrosome breakage during DC migration along a three-way path junction in the presence of 5 μM GSK-626616 or control (DMSO), or upon cNAP1 deletion. (**C**) As in (B), quantifying centriolar distances before, during, and after migration through path junctions of cells that pass the junction either with two simultaneous explorative protrusions or DCs that immediately decide for one path alternative with one protrusion. (**D**) Immunofluorescence staining of CETN2-GFP (red, arrows)–expressing DCs upon migration through bead obstacles underneath an agarose layer. DAPI (blue), anti–α-tubulin (black and magenta), and phalloidin (black and yellow) visualize the nucleus, the microtubule, and the F-actin cytoskeleton, respectively. (**E**) As in (B), but in co-presence of 50 nM Latrunculin A, 25 μM para-nitro-blebbistatin, or control (DMSO). (**F**) Immunofluorescence of Dyrk3-inhibited (5 μM GSK-626616) CETN2-GFP (red, arrows)–expressing DCs upon migration through bead obstacles underneath an agarose layer showing representative examples of intact and fractured centrosomes. Anti–α-tubulin (black) and DAPI (blue) visualize the microtubule cytoskeleton and the nucleus, respectively. (**G** to **J**) Quantification of microtubule aster formation (G), the microtubule anchoring protein ninein (H), the microtubule nucleator γ-tubulin (I), and Akna (J) upon breakage of the centrosome. Time is indicated as hours:minutes:seconds.

The centriolar pair in the centrosome is well-known to be connected by a protein-based linker that involves cNAP1 (*33*), and RPE1 cells with a knockout in cNAP1 migrate slower on 2D substrates (*8*). To identify whether this known linker mechanism is involved in maintaining centrosome integrity under forces during navigational pathfinding, we generated a conditional knockout of cNAP1 (*CEP250*) in CETN2-GFP–expressing DCs (fig. S6, A to F). Analysis revealed increased centriolar distances and centrosomal fracturing rates when cells transitioned through path junctions (Fig. 5, A and B). To investigate whether this was due to competing protrusions, we analyzed the centrosomal fracturing rate and only observed increased fracturing rates when cells explored their path with competing protrusions (Fig. 5C). When we targeted Dyrk3 and cNAP1 simultaneously, centrosome fracturing rates were not significantly increased compared to their individual targeting, indicating their function in the same protective pathway (Fig. 5B). Together, these data establish that the centrosome is prone to breakage by migratory forces, requiring protective mechanisms mediated by the known centriolar linker cNAP1 and the here identified role of Dyrk3 in centrosome cohesion.

### Forces causing centrosome fracturing

To identify the forces causing centrosome fracturing, we aimed to disentangle extracellular from intracellular forces. First, we exposed cells to environmental confinement by squeezing the cells between two layers (*34*), causing only occasional centrosome fracturing in a Dyrk3-dependent manner (fig. S4, B and C). Similarly, we detected occasional centrosome deformations during cellular squeezing through narrow 2-μm pores, while centrosome stretching already occurred during translocation through wider 3-μm pores when Dyrk3 was nonactive (fig. S7, A to D, and movie S4). These data suggest that mechanical stresses from the microenvironment cause centrosome deformations. Nevertheless, centrosome fracturing was more frequent during cellular pathfinding, suggesting that intracellular forces from competing protrusions may cause larger centrosome deformations. To combine both environmental confinement and competing protrusions, we embedded micrometer-sized beads as path obstacles between two layers, generating confined cells that have to navigate (fig. S5, A to C) and observed high rates of centrosome fracturing when Dyrk3 was nonfunctional (fig. S4, D and E). These findings suggest that, while cellular confinement within the microenvironment can lead to centrosome fracturing, fracturing occurs more frequently due to intracellular forces derived from competing protrusions.

To identify the source of intracellular forces causing centrosome deformations and fracturing, we focused on the actomyosin cytoskeleton, as actin is known to locate at the centrosome of lymphocytes (*35*, *36*) and DCs (*11*) (Fig. 5D) and as actomyosin forces contribute to centriole separation during cell cycle progression (*37*). To address whether actomyosin forces cause centrosome fracturing, we exposed DCs to path junctions and low doses of the actin inhibitor Latrunculin. This allowed functional migration (*18*) but prevented centrosome fracturing when Dyrk3 is nonactive (Fig. 5E, fig. S8A, and movie S5). Similarly, inhibition of myosin reduced the rates of centrosome fracturing (Fig. 5E, fig. S8A, and movie S5). To corroborate this finding, we inhibited actin and myosin in DCs with conditional knockouts of the known centriolar linker protein cNAP1 and observed reduced centrosome fracturing during pathfinding upon actin inhibition but, to a lesser extent, upon myosin inhibition (Fig. 5E and fig. S8B). When we inhibited Arp2/3 or formin actin nucleators, centrosome fracturing in conditional cNAP1 knockouts and Dyrk3-inhibited cells was reduced upon formin inhibition (fig. S8C). Moreover, using the PCM marker Pericentrin, we observed diminished PCM deformations upon actin inhibition (fig. S8, C and D). Thus, forces from the actomyosin cytoskeleton lead to centrosome fracturing during cellular pathfinding.

### Centrosome fracturing generates coexisting MTOCs

Experimental centriole depletion often results in mild cellular phenotypes (*38*), including migration without centrioles, as other organelles like Golgi membranes (*39*) can function as alternative microtubule-organizing centers (MTOCs) (*4*, *40*, *41*), compensating for centriole-based MTOCs. Our findings on centrosome fracturing raised the possibility that the consequences of fractured centrosomes are entirely different from experimental centrosome depletion. To investigate this hypothesis, we visualized the microtubule cytoskeleton, unexpectedly observing separate MTOCs around individual centrioles upon centrosome fracturing following Dyrk3 inhibition (Fig. 5, F and G, and fig. S9A) and in the rare cases of centrosome fracturing in WT cells when centrosomal shielding mechanisms are in place (fig. S10, A and B). In contrast, intact centriole pairs formed single MTOCs as revealed by stimulated emission depletion (STED) microscopy (fig. S9, B and C), which asymmetrically localized the microtubule-anchoring protein ninein (*7*, *42*), and efficiently nucleated microtubules tracked by EB3-positive plus ends (fig. S9, D to F), even when Dyrk3 was inhibited (Fig. 5F; fig. S9, D to F; and movie S6).

These results suggested that broken centrosomes can form coexisting functional MTOCs within one cell. To confirm this finding, we engineered DCs to stably express CETN2-GFP and EB3-mCherry as centriolar and MTOC markers (*18*, *24*), identifying by live-cell imaging two coexisting MTOCs in most cells upon centrosome fracturing (fig. S10, C and D). Microtubules detached only occasionally (fig. S9G) and ninein mostly localized to both centrioles upon fracturing (Fig. 5H and fig. S11A), suggesting functional microtubule anchoring. Similarly, the microtubule nucleator γ-tubulin and the centrosomal protein AKNA localized around both centrioles after fracturing (Fig. 5, I and J, and fig. S11, B and C). Thus, centrosome fracturing leads to the emergence of functional coexisting MTOCs within single cells.

### Centrosome fracturing impedes cellular navigation

**Fig. 6.**
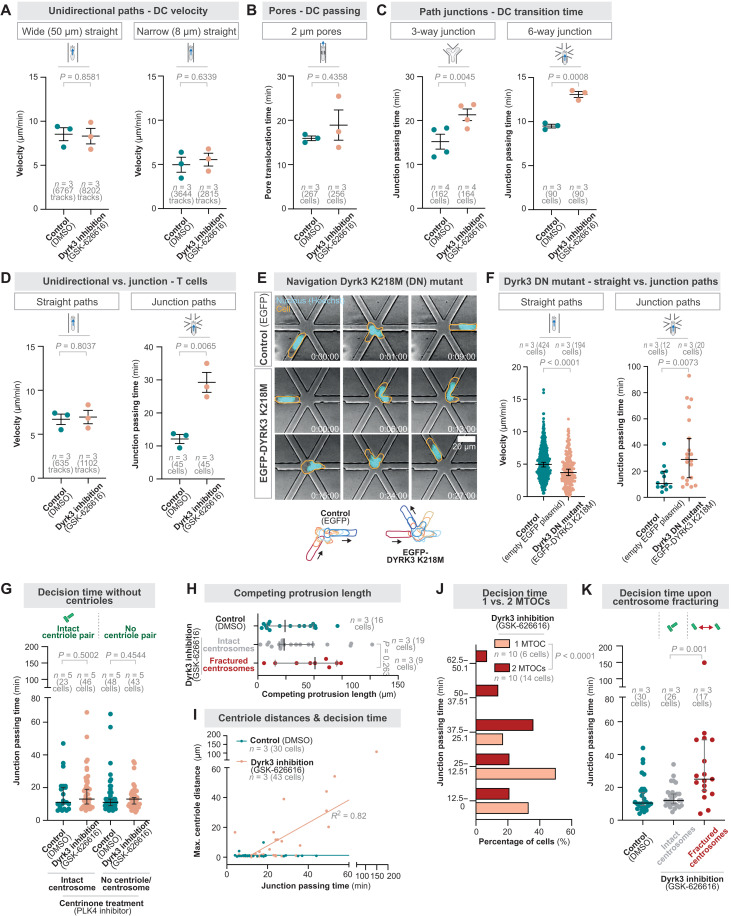
Centrosome fracturing impedes cellular navigation. (**A** to **C**) DC migration velocity along unidirectional paths (A), 2-μm pores (B), and path junctions (C) in the presence of 5 μM GSK-626616 or DMSO (control). (**D**) Jurkat T cell migration velocity along unidirectional paths or six-way path junctions in the presence of 5 μM GSK-626616 or DMSO (control). (**E**) Representative EGFP-Dyrk3 K218M or EGFP expressing Jurkat T cells migrating through a six-way path junction. Time is indicated as hours:minutes:seconds. (**F**) Jurkat T cell migration velocity along unidirectional paths or passing time in six-way path junctions of cells expressing a dominant-negative (DN) EGFP-Dyrk3 K218 mutant or the corresponding empty EGFP plasmid. (**G**) Path decision time of centriole-depleted and non-centriole–depleted CETN2-GFP DCs through six-way decision points. Note that centrinone treatment results in a heterogeneous cell population with variable centriole numbers (see Materials and Methods for details). (**H**) Length of the major competing protrusion of DCs with fractured and non-fractured centrosomes migrating through six-way junctions. (**I**) Correlation of the cellular decision time [as in (K)] with the maximal distance between individual centrioles. (**J**) Path decision time of CETN2-GFP EB3-mCherry–expressing DCs with fractured centrosomes forming one or two MTOCs along six-way path junctions in the presence of 5 μM GSK-626616. (**K**) Path decision time of CETN2-GFP DCs with fractured and non-fractured centrosomes through six-way decision points.

Given the importance of single MTOCs as a steering organelle during cellular locomotion (*12*, *24*), we investigated the functional consequences of coexisting MTOCs for moving cells. Rendering the centrosome prone to fracturing by impairing Dyrk3 activity resulted in unaffected migration along straight paths and through narrow 2-μm constrictions (Fig. 6, A and B; fig. S12, A to C; and movie S7). This is consistent with microtubules being dispensable for unidirectional movement, as the actin cytoskeleton is the driving force for locomotion (*9*, *43*). In contrast, DCs moving through micro-channels with either three-way or six-way junctions needed longer to perform path decisions in the presence of GSK-626616 (Fig. 6C; fig. S12, D and E; and movie S7). These data suggested that the emergence of coexisting MTOCs upon centrosome fracturing impairs pathfinding. To test generality, we measured the migration properties of T cells upon the expression of the kinase-dead point mutant of DYRK3 or Dyrk3 inhibition, showing delayed pathfinding, while migration along straight paths was unaffected (Fig. 6, D to F; fig. S13, A to C; and movie S8).

In line with a pathfinding phenotype, DCs and Jurkat T cells that either express the kinase-dead point mutant of DYRK3 or are inhibited by GSK-626616 showed elongated shapes (fig. S14, A to C). To exclude a cell polarity defect due to Dyrk3 nonfunctionality independent of centrosome fracturing, we measured the configuration of the nucleus-MTOC axis as a polarity marker, revealing a normal nucleus-forward configuration (fig. S15, A and B, and movie S9). To confirm a normal polarity in cells with nonactive Dyrk3 and intact centrosomes, we genetically engineered DCs to express the PIP_3_/PI(3,4)P_2_ sensor PH-Akt, a well-established polarity marker (*44*). Directionally migrating cells showed a preferentially forward localization of PH-Akt, which remained unchanged upon impairing Dyrk3 activity (fig. S15, C to E, and movie S9). Together, these data show that altering Dyrk3 activity does not impair cellular polarity as long as the centrosome remains intact, and, therefore, only one MTOC is present.

Thus, we tested the direct consequences of the emergence of two MTOCs upon centrosome fracturing by live-cell imaging during path decisions. This revealed that specifically cells with fractured centrosomes had longer competing protrusions (Fig. 6H) and required more time for productive pathfinding (Fig. 6K), where even the delay of passage correlated with the separation distance between fractured centrosome parts (Fig. 6I). In a cell race–like scenario, where cells must continuously perform path decisions like in tissues, cells with broken centrosomes and multiple MTOCs migrated substantially shorter distances than cells with intact centrosomes (fig. S16, A to C). Further, live-cell imaging of emerging MTOCs upon centrosome fracturing revealed delayed path decisions specifically when broken centrosomes formed competing MTOCs (Fig. 6J). Thus, only the emergence of coexisting MTOCs impairs cellular pathfinding. In contrast, cells without any centriole but with a single functional MTOC (fig. S17, B and C) (*45*), generated by the PLK4 inhibitor centrinone (*46*), showed normal migration during pathfinding, even if Dyrk3 is nonactive (Fig. 6G, fig. S17A, and movie S10). Thus, while cells are effective in compensating for experimentally induced centriole depletion, they must protect their centrosome from forces to prevent fracturing and the emergence of competing MTOCs. Together, these findings discover centrosome deformations that can lead to centrosome fracturing, which impedes cell functionality by generating coexisting MTOCs and causes entanglement in the microenvironment.

## DISCUSSION

How membraneless organelles (*47*) respond to mechanical forces and withstand them is largely unknown. The centrosome is a cellular membraneless organelle (*14*, *48*–*50*) that undergoes a highly regulated and timed separation during cell division. While the absence of a surrounding membrane is critical for this controlled separation at each cell division, our findings discover that centrosomes are exposed and susceptible to deformations in nondividing cells due to both extracellular and intracellular forces during the migration of cells. Specifically, we identify that forces are transmitted to the centrosome during cell migration through confining or complex environments during cellular squeezing and navigational pathfinding, where, in particular, forces from the actin cytoskeleton deform and even fracture the centrosome. This indicates a mechanical nature of centrosome fracturing and that actin forces act not only as intracellular organelle “movers” but also as “destroyers.”

Our data not only discover that centrosomes are exposed to forces but reveal that the integrity of the centrosome is protected by the kinase Dyrk3. However, whether the previously described function of Dyrk3 as a phase condensate dissolvase is involved in this mechanism remains to be identified, as well as the precise mechanisms of actin-based force transmission to the centrosome that may involve either actin directly at the centrosome or a connection from force-generating actin pools at cellular protrusions to the centrosome. Our data further identify that the previously known centriolar linker protein cNAP1 is required to maintain centrosome cohesion when cells are exposed to forces experienced during cell migration through 3D environments. While a knockout of cNAP1 leads to viable mice [but causes male infertility; (*51*)], our data in DCs indicate an important function of cNAP1 given that the low knockout rates of around 20% on the allelic level in the used conditional knockout system correlate with a similar rate of centrosome fracturing at path junctions, potentially indicating a critical role of cNAP1 in DC-mediated immune responses and more broadly in a functional immune system.

Our findings further establish that these centrosomal shielding mechanisms protect cells from the detrimental formation of multiple MTOCs around broken centrosome fragments. These MTOCs around individual centrioles nucleate microtubules and show localization of the microtubule anchoring protein ninein, indicating proper MTOC functionally. Our data show that, specifically, the emergence of more than one MTOC causes defects in cellular decision-making at path junctions, leading to the entanglement of motile amoeboid and mesenchymal cells in the matrix upon centrosome fracturing.

Overall, our findings identify the importance of maintaining centrosome integrity for cellular functionality and tightly regulating MTOC numbers. Further, they indicate that cellular behaviors resulting from centrosome breakage differ from those arising from experimental centriole depletion. Beyond its relevance for cells moving in multicellular organisms, such as during immune surveillance and cancer cell motility, our findings suggest the fundamental concept that cells have to preserve the integrity of the centrosome and other membraneless organelles by shielding mechanisms whenever they experience mechanical forces, such as during development, tissue homeostasis, or disease. Further, these findings add to the emerging concept that intracellular organelles are not only actively positioned (*18*, *52*–*54*) but also experience physical forces (*55*, *56*), requiring their shielding to maintain cellular functionality.

## MATERIALS AND METHODS

### Cell culture

All cells were grown and maintained at 37°C in a humidified incubator with 5% CO_2_. Jurkat T cells were cultured in R10 medium at a cell density of 0.1 × 10^6^ to 1.5 × 10^6^ cells/ml. 3T3 fibroblasts were cultured in Dulbecco’s modified Eagle’s medium (DMEM; 31885, Gibco) supplemented with 10% fetal calf serum (FCS), penicillin (100 U/ml), streptomycin (100 mg/ml), and 0.1 mM 2-mercaptoethanol (all Gibco) at 70 to 80% confluency. DCs were differentiated either from bone marrow isolated from male C57Bl6/J wild-type mice (aged 8 to 12 weeks) or from Hoxb8 (*57*) precursor cell lines [CETN2-GFP (*11*), EB3-mCherry (*12*), Lifeact-GFP and EMTB-mCherry, CETN2-GFP and EB3-mCherry, CETN2-GFP and PH-Akt-dTomato, PCNT-dTomato and CETN2-GFP and indCas9 sgC-NAP1]. Cells were cultured in R10 medium [RPMI 1640 and 10% FCS, 2 mM l-glutamine, penicillin (100 U/ml), streptomycin (100 mg/ml), and 0.1 mM 2-mercaptoethanol, all Gibco] supplemented with 10% granulocyte-macrophage colony-stimulating factor hybridoma supernatant. On differentiation days 3 and 6, fresh medium was added. CETN2-GFP and indCas9 sgC-NAP1 Hoxb8 cells were grown and maintained in a conditioned medium supplemented with blasticidin (10 μg/ml) and puromycin (3 μg/ml). Cas9 expression was induced by the addition of doxycycline (1 μg/ml) on differentiation day 0 and continued by the addition of doxycycline (2 μg/ml) on days 3 and 6. For migration experiments, either fresh or thawed DCs (differentiation day 8) were stimulated by adding lipopolysaccharide (200 ng/ml; *Escherichia coli* O26:B6; MilliporeSigma) for 24 hours to induce cell maturation. For centriole depletion experiments, differentiating DCs were treated with a final concentration of 500 nM centrinone B [Bio-Techne; dissolved in dimethyl sulfoxide (DMSO)] on differentiation days 2, 3, 6, 7, and 8. As described previously (*46*), PLK4 inhibition upon centrinone B treatment prevents the formation of new daughter centrioles, while preexisting centrioles are further distributed during cell division. Consequently, when added over multiple rounds of cell division, centrinone B treatment ultimately results in a mixed cell population composed of cells with no, one, two, or even multiple centrioles at the same time (*11*, *45*, *46*).

### Mice

All animals were housed in the Core Facility Animal Models at the Biomedical Centre (Ludwig-Maximilians-Universität), and animal procedures and experiments were in accordance with the ministry of animal welfare of the region of Oberbayern and with the German law of animal welfare.

### Flow cytometry analysis

DC maturation was routinely checked for surface expression of CD11c and major histocompatibility complex class II (MHCII). After Fc receptor blockage using an anti-mouse CD16/32 antibody (14-0161-85, Invitrogen) diluted in fluorescence-activated cell sorting (FACS) buffer [1% bovine serum albumin (BSA) and 2 mM EDTA in phosphate-buffered saline (PBS)], cells were stained with anti-mouse CD11c and anti-mouse MHCII antibodies (17-0114-82 and 48-5321-82, both Invitrogen). Flow cytometry analysis was performed on a CytoFLEX S flow cytometer (Beckman Coulter).

### Transgenic cell lines and transgene delivery

#### Generation of inducible Cas9 hematopoietic progenitor cell line

Hematopoietic progenitor cell lines were generated by retroviral delivery of an estrogen-regulated form of *Hoxb8* as described recently (*57*, *58*). Briefly, the bone marrow of 8- to 12-week-old CETN2-GFP–expressing mice was isolated and retrovirally transduced with an estrogen-regulated form of the HOXB8 transcription factor. Cells were cultured in an estradiol-containing medium to allow expression of HOXB8. To generate an inducible Cas9 cell line, a lentiviral approach was applied.

For lentivirus production, 10^6^ human embryonic kidney 293 FT cells were seeded. A plasmid mix containing psPAX2 (packaging plasmid, gift from D. Trono; Addgene, plasmid no. 12260), pMD2.G (envelope plasmid, gift from D. Trono; Addgene, plasmid no. 12259), and indCas9-Blast (Horizon Discovery, CAS 11229) (2:3:4 mass ratio) in a total concentration of 8 ng/ml was transduced. After 5 hours, the medium was changed with prewarmed DMEM. Lentivirus was harvested 48 hours later by collecting and filtering the supernatant through a 0.45-μm filter. For lentiviral transduction, a cell suspension of HOXB8 cells at 0.3 × 10^6^/ml supplemented with polybrene (8 μg/ml; TR-1003-G, Merck Millipore) was prepared, along with different virus concentrations. Cells were spinfected by centrifuging at 1500*g* for 90 min at 32°C. After spinfection, 1.5 ml of fresh medium was added. After 1 day, the medium was renewed to reduce polybrene. Selection with blasticidin (10 μg/ml) was initiated after 3 days, and, after 7 days, cells with a multiplicity of infection of 0.04 were selected for further use.

#### CRISPR-knockout generation

To generate C-NAP1–specific knockouts of immortalized hematopoietic progenitor reporter cell lines, a lentiviral CRISPR-Cas9 approach was applied. Single-guide RNAs were designed using CHOPCHOP. Top- and bottom-strand primers were designed, containing distinct overhangs (top primer, 5′-CACCGGTGCATGAGCGTGCCGACGA-3′; and bottom primer, 5′-AAACTCGTCGGCACGCTCATGCACC-3′) needed for cloning and U6 promoter-dependent transcription. Top- and bottom-strand primers were resuspended to a concentration of 100 μM, annealed, and cloned into the CROP-Seq_Guide_Puro plasmid (a gift from C. Bock; Addgene, no. 86708) (*59*). Virus production and infection were carried out as described above. Seventy-two hours postinfection, cells were washed to remove the remaining viruses, and a selection medium containing puromycin dihydrochloride (3 μg/ml) was added.

To determine indel frequency, 1 × 10^5^ cells from each condition were harvested, resuspended in 75 μl of cell lysis buffer (25 mM NaOH and 0.2 mM EDTA), and incubated at 95°C for 30 min. Following neutralization with 75 μl of cell neutralization buffer (40 mM tris-HCl, pH 5.5), 20 μl of neutralized cell lysate was used to perform DNA amplification with the Platinum Hot Start PCR Mix (13001013, Thermo Fisher Scientific) according to the manufacturer’s instructions for 34 cycles (forward primer, 5′-ACACTCTTTCCCTACACGACGCTCTTCCGATCTCTGGCACTCTACCTCCTTCTCCT-3′; and reverse primer, 5′-TGACTGGAGTTCAGACGTGTGCTCTTCCGATCTGGTGCTGCAGAAGGAAAGGATTC-3′; annealing temperature, 55°C). The first polymerase chain reaction (PCR; 1 μl) was used for the secondary barcoding PCR (M0541S, Thermo Fisher Scientific). After column purification and NanoDrop quantification, samples were sequenced using an Illumina Miseq machine. Afterward, data were analyzed using the Outknocker online tool (www.outknocker.org/outknocker2.htm) (*60*).

#### Fluorescent reporter constructs

Generation of a N-terminal dTomato fusion construct of Pericentrin was performed by amplifying Pericentrin from a red fluorescent protein (RFP)–Pericentrin encoding plasmid (a gift from M. Heuze) (*61*) using a Bsr GI restriction site containing forward (5′-CGAGCTGTACAAGGGTGGTTCTGGTGAGCAAAAGC-3′) and an Eco RI restriction site containing reverse (5′-GGAACGAATTCCTACTGTTTAATCATCGGGTGGC-3′) primer pair. N-terminal dTomato fusions constructs of PH-Akt were generated by amplifying PH-Akt from a PH-Akt-GFP encoding plasmid (gift from T. Balla;

Addgene, no. 51465) (*44*) using a Bsr GI restriction site containing forward (5′-CGAGCTGTACAAGGGTGGTTCTGGTAGCGACGTGG-3′) and an Eco RI restriction site containing reverse (5′-GGAACGAATTCCTAGGTGGCGACCGGTGG-3′) primer pair. After Bsr GI and Eco RI digestion, DNA fragments encoding Pericentrin and PH-Akt were cloned into a pLenti6.3/TO/V5-DEST backbone containing dTomato (a gift from E. Snaar-Jagalska; Addgene, plasmid no. 106173) using the Quick Ligation Kit. The correct sequence and orientation of clones were verified by sequencing (Eurofins).

Generation of the pmaxGFP construct was performed by amplifying GFP from pcDNA5/FRT/TO-GFP (a gift from H. Kampinga; Addgene, plasmid no. 19444) using an Eco RI restriction site containing forward (5′-TAAGCAGAATTCATGGTGAGCAAGGGCGAGGA-3′) and a Bam HI restriction site containing reverse (5′-TGCTTAGGATCCCTACTTGTACAGCTCGTCCATGC-3′) primer pair. Generation of pmax-GFP-Dyrk3-K218M constructs was performed by amplifying GFP-Dyrk3-K218M from pcDNA5-Dyrk3-K218M-GFP (a gift from L. Pelkmans and D. Dormann) using an Eco RI restriction site containing forward (5′-TAAGCAGAATTCATGGTGAGCAAGGGCGAGGA-3′) and a Bam HI restriction site containing reverse (5′-TGCTTAGGATCCCTAGCTAATCAGTTTTGGCAATA-3′) primer pair. After Eco RI and Bam HI digestion, DNA fragments were subcloned into a pmaxCloning (Lonza, catalog no. VDC-1040) using the Quick Ligation Kit. The correct sequence and orientation of clones were verified by sequencing (Eurofins).

The fluorescent plasmid DNA reporter constructs coding for EB3-mCherry and EMTB-mCherry were a gift from M. Sixt (Institute of Science and Technology, Vienna, Austria). M. Götz (Institute for Physiological Genomics, Munich, Germany) provided Akna constructs. CEP120 constructs were a gift from T. Tang (Addgene, no. 50382) (*62*).

#### Transgene delivery of fluorescent reporter constructs

For stable expression, lentiviral transduction of Hoxb8 cells was performed. Lentivirus was produced by cotransfecting LX-293 cells with the respective fusion construct encoding plasmid in combination with pMD2.G (a gift from D. Trono; Addgene, plasmid no. 12259) and psPAX2 (a gift from D. Trono; Addgene, plasmid no. 12260). The supernatant of virus-producing cells was harvested 48 hours after transfection and either used directly for lentiviral transduction or stored at −80°C. After lentiviral infection in the presence of polybrene (3 μg/ml), Hoxb8 cells were selected for stable virus insertion using blasticidin (10 μg/ml) for at least 1 week. Following cell expansion, Hoxb8 cells expressing fluorescent reporter constructs were sorted using FACS on a FACSAriaFusion (BD Biosciences) equipped with four lasers (405, 488, 561, and 640 nm).

For transient expression of fluorescent reporter constructs, Jurkat T cells and 3T3 fibroblasts were electroporated with plasmids encoding the respective constructs 16 or 48 hours before the experiment, respectively, using the Neon Transfection system (Invitrogen) with three pulses at 1600 V for 10 ms each.

Dyrk3 localization was analyzed by co-electroporating Jurkat T cells with pcDNA5-Dyrk3-WT-GFP (*22*) (a gift from L. Pelkmans and D. Dormann) and mCherry-Centrin2-N-10 (a gift from M. Davidson; Addgene, plasmid no. 55018). The next day, cells were injected in an under-agarose migration assay as described below, and mCherry^+^ GFP^+^ cells were imaged.

For validation of Dyrk3 inhibition–mediated effects on migration, Jurkat T cells were transfected either with pcDNA5/FRT/TO-GFP (*63*) (a gift from H. Kampinga; Addgene, plasmid no. 19444) as control or pcDNA5-Dyrk3-K218M-GFP (*22*) (a gift from L. Pelkmans and D. Dormann) encoding for a dominant-negative point-mutant Dyrk3, respectively. The next day, cells were prepared for FACS on a FACSAriaFusion (BD Biosciences) equipped with four lasers (405, 488, 561, and 640 nm). Live GFP^+^ cells were directly sorted into R10 medium buffered with 25 mM Hepes. After 1 hour of recovery in the incubator, cells were used for downstream migration assays as described below.

For validation of Dyrk3 inhibition–mediated effects on mesenchymal migration, 3T3 fibroblasts were electroporated either with pmax-GFP as control or pmax-Dyrk3-K218M-GFP, respectively. Afterward, cells were directly used for downstream migration assays as described below.

### Micro-fabricated devices

Micro-fabricated devices were prepared as described previously (*64*). Briefly, microstructures were replicated from custom-made wafers produced by photolithography or epoxy replicates as templates, with defined width, height, length, and pore sizes. The height of the microstructures ranged between 4 and 5 μm to allow cell confinement from top and bottom. Wide straight channels had a width of 50 μm. Narrow straight channels, channels with constrictions, three-way pathfinding channels, and six-way pathfinding channels had a width of 8 μm, thereby confining cells from all sides. Distance between two six-way crossings was 90 μm. The pore size of micro-channels with constrictions was 2, 3, or 4 μm as indicated. 3D micropillars for Jurkat T cells had a height of 5 μm, with pillars of 20-μm diameter and distance in wide mazes and with pillars of 7-μm diameter and 4-μm distance in narrow mazes. 3D micropillars for 3T3 fibroblasts had a height of 7.6 μm with pillars of 7-μm diameter and 6- or 10-μm distance between pillars.

Polydimethylsiloxane (PDMS; 10:1 mixture of Sylgard 184, Biesterfeld) was added onto the template structures to generate replica of the microstructures. Air bubbles were removed with a desiccator. After solidification at 80°C overnight, PDMS was carefully removed from the templates and cut into pieces according to the respective design size. Holes for cell and chemokine loading were punched. Using a plasma cleaner, the PDMS device was bonded to clean glass coverslips. PDMS devices were then placed at 120°C for 10 min, followed by overnight incubation at 80°C to permanently bond them to the glass surface.

### Live-cell migration assays

For live-cell imaging, cell nuclei were visualized by preincubating cells for at least 30 min with NucBlue (Invitrogen) according to the manufacturer’s instructions, followed by washing. For pharmacological inhibition experiments, final concentrations of 1 to 10 μM GSK-616626 (Tocris; dissolved in DMSO), 10 to 100 μM harmine (Sigma-Aldrich; dissolved in DMSO), 50 nM Latrunculin A (Sigma-Aldrich; dissolved in DMSO), 50 μM CK666 (Sigma-Aldrich; dissolved in DMSO), 15 μM Smifh2 (Bio-Techne; dissolved in DMSO), and 25 μM para-nitro-blebbistatin (Motorpharma; dissolved in DMSO) were used as indicated. Control samples were treated with DMSO in the corresponding dilution.

#### Micro-channel migration assays

Before the experiment, PDMS devices were flushed with phenol-free R10 medium supplemented with 50 μM l-ascorbic acid (MilliporeSigma) and inhibitors if needed according to the experimental setup. After incubation at 37°C and 5% CO_2_ in a cell culture incubator for at least 1 hour, devices were used for downstream experiments. Then, CCL19 (0.625 μg/ml; DCs) or CXCL12 (1.25 μg/ml; T cells) was loaded into the chemokine loading hole to establish a chemokine gradient, followed by addition of 0.3 × 10^5^ to 0.5 × 10^5^ cells into the second loading hole. For 3T3 fibroblast micropillar assays, 3 × 10^5^ cells were loaded into one loading hole 24 hours before imaging for Dyrk3 inhibition experiments and 6 hours for Dyrk3-K218M experiments. 3T3 fibroblasts migrated undirectedly in the absence of a chemotactic gradient at 37°C and 5% CO_2_ for 24 hours.

#### Under-agarose migration assays

Under-agarose migration assays without bead obstacles were prepared as described previously (*65*). Briefly, 1% agarose was prepared by mixing 4% UltraPure agarose (Invitrogen) in sterile water with 55°C prewarmed phenol-free RPMI 1640 (Gibco) supplemented with 20% FCS and 1× Hanks’ balanced salt solution (pH 7.3) in a 1:3 ratio. For experiments including inhibitors, the 1% agarose mixture was let cool down to 37°C before adding the inhibitor to the respective final concentration. The agarose was poured into imaging-suitable eight-well slides (ibidi), polymerized for 1 hour at room temperature, and then transferred to the incubator for 1 hour for equilibration. For under-agarose migration assays including bead obstacles, eight-well slides were pre-coated with Polybeads (6-μm diameter; 07312-5, Polysciences). The Polybeads were washed and resuspended in phenol-free R10 medium. After activating the well glass surface with oxygen plasma, beads were added for coating. Then, the agarose mixture was added after additional 30 min to ensure stable bead attachment. For both, under agarose with and without bead obstacles, 2-mm-wide holes were generated using tissue biopsy punchers after agarose solidification and equilibration. Then, CCL19 (2.5 μg/ml; DCs) or CXCL12 (5 μg/ml; T cells) in phenol-free R10 was loaded as chemotactic stimulus. Cells (0.2 × 10^5^) were injected between the glass surface and agarose layer in a distance of 2 to 3 mm to the chemokine loading hole. For live-imaging, assays were placed in the incubator for 1 hour to allow induction of directional migration toward the chemokine source before imaging.

#### Collagen migration assays

Collagen migration assays were performed as described previously (*64*, *66*). Briefly, for DC collagen migration assays, PureCol bovine collagen (Advanced BioMatrix) in 1× minimum essential medium (Sigma-Aldrich) and 0.4% sodium bicarbonate (Sigma-Aldrich) was mixed with 3 × 10^5^ cells in R10 at a 2:1 ratio, resulting in gels with a collagen concentration of 1.7 mg/ml, forming pore sizes ranging from 1- to 5-μm diameter (*24*). Collagen-cell mixtures were cast in custom-made migration chambers with a diameter of 18 mm and a height of ~1 mm. After polymerization of collagen fibers at 37°C and 5% CO_2_ in a cell culture incubator for 75 min, 80 μl of CCL19 (0.625 μg/ml; 440-M3-025, Bio-Techne) was added to the top of the chamber. For Jurkat T cell collagen migration assays, PureCol stock solution was diluted with PBS, resulting in a final collagen concentration of 1.3 mg/ml mixed with 2 × 10^5^ cells in R10 at a 2:1 ratio. After polymerization at 37°C and 5% CO_2_, in a cell culture incubator for 75 min, 80 μl of CXCL12 (1.25 μg/ml; 350-NS-050, Bio-Techne) was added to the top of the chamber. For inhibition experiments, inhibitors were added to the collagen-cell mixture as well as to the chemokine solution at the indicated concentrations.

### Whole-mount ear skin immunohistology

Preparation of ear sheet staining was carried out as described recently (*11*). Briefly, the ears of 5- to 8-week-old C57BL/6J mice were cut off and separated into dorsal and ventral sheets using forceps. Ventral ear sheets were further placed in a 24-well plate upside down on complete medium [RPMI 1640 supplemented with 10% FCS, 2 mM l-glutamine, penicillin (100 U/ml), streptomycin (100 μg/ml), and 50 μM ß-mercaptoethanol; all Gibco] and incubated for 24 hours. Afterward, samples were fixed with 4% paraformaldehyde (PFA) and immersed in 0.2% Triton X-100 for 30 min for antibody staining. Dermal DCs were visualized using a biotinylated antibody against MHCII (M5/114.15.2), which binds streptavidin. Vessels were stained with LYVE1 [rat anti-mouse Lyve-1 (ALY7)] and anti-rat antibody coupled to Alexa Fluor 488 overnight at room temperature. Primary antibodies were diluted in 1% BSA in a ratio of 1:400 for MHCII-biotin and 1:200 for LYVE1 and applied after 1 hour of incubation with 1% BSA. Because both primary antibodies were of rat origin, the MHCII antibody was initially blocked with streptavidin-Cy3 (diluted in 1% BSA, 1:400) for 1 hour, before the secondary antibody [donkey anti-rat Cy3 AffiniPure immunoglobulin G (H+L); diluted in 1% BSA, 1:400] was added. Ears were conserved in a nonhardening mounting medium without 4′,6-diamidino-2-phenylindole (DAPI; Invitrogen) and stored at 4°C in the dark.

### Immunofluorescence stainings

For immunofluorescence stainings, under-agarose migration assays were prepared as described above. Following cell migration for 2 hours, 3.7% PFA (diluted in PBS) prewarmed to 37°C was added on top of the agarose and incubated for 1 hour at 37°C and 5% CO_2_. After fixation, the agarose block was carefully removed, and cells were washed with PBS. Following permeabilization with 1× SAPO buffer (0.2% BSA and 0.05% saponin diluted in PBS) for 30 min, blocking was performed with 5% BSA (diluted in 1× SAPO). Primary antibodies were incubated overnight at 4°C [rat anti–α-tubulin, 2 μg/ml (MA1-80017, Invitrogen); rabbit anti-ninein, 0.25 μg/ml (PA5-82224, Invitrogen); mouse anti–γ-tubulin, 4.25 μg/ml (T6557, Sigma-Aldrich); mouse anti-Akna, 1:25, in house-production, gift from M. Götz (Institute for Physiological Genomics, Munich, Germany); rabbit anti-Ccdc88b, 0.8 μg/ml (HPA026652, Atlas Antibodies); and rabbit anti-Akna, 1 μg/ml (HPA052367, Atlas Antibodies); all diluted in 1× SAPO]. The next day, samples were washed with PBS and stained with secondary antibodies [goat anti-rat Alexa Fluor Plus 647, 4 μg/ml (A48265, Invitrogen); donkey anti-rabbit Alexa Fluor Plus 647, 4 μg/ml (A32795, Invitrogen); goat anti-mouse Alexa Fluor 555, 2 μg/ml (ab150114, Abcam); and phalloidin Alexa Fluor Plus 647, 165 nM (A30107, Invitrogen); all diluted in 1× SAPO] and DAPI (1:1000; Thermo Fisher Scientific) at room temperature for 1 hour. After washing with PBS, cells were mounted using Fluoromount-G (Invitrogen).

For super-resolution imaging of microtubules, under-agarose migration assays were performed as described above. After cell migration for 2 to 4 hours, 3.7% PFA and 0.5% glutaraldehyde diluted in microtubule-stabilizing buffer [MTSB; 80 mM K-Pipes (pH 6.8), 1 mM MgCl_2_, and 5 mM EGTA in ddH_2_O, pH adjusted to 6.8 with KOH] prewarmed to 37°C was added on top and incubated for 30 min at 37°C and 5% CO_2_. After careful removal of the agarose block, quenching solution (0.1% NaBH_4_ diluted in PBS) was added to reduce glutaraldehyde-induced background fluorescence and incubated for 8 min at room temperature. Following permeabilization, blocking, and anti–α-tubulin primary antibody incubation as described above, samples were washed with PBS and stained with the secondary antibody (goat anti-rat Alexa Fluor Plus 594, 4 μg/ml; A11007, Invitrogen; diluted in 1× SAPO) at room temperature for 1 hour. Subsequent washing and mounting were performed as described above.

For immunofluorescence staining of DCs within 3D micropillars, micro-channel migration assays were performed as described above. After migration for 4 to 6 hours, 3.7% PFA and 0.5% glutaraldehyde diluted in MTSB [80 mM K-Pipes (pH 6.8), 1 mM MgCl_2_, and 5 mM EGTA in ddH_2_O, pH adjusted to 6.8 with KOH] prewarmed to 37°C were first added to one loading hole and incubated for 30 min at 37°C and 5% CO_2_. After 30 min, fixation solution was added to the second loading hole and incubated for additional 30 min at 37°C and 5% CO_2_. Afterward, quenching was performed by adding quenching solution (0.1% NaBH_4_ diluted in PBS) to the first loading hole, and, following incubation for 10 min at room temperature, quenching solution was added to the second loading hole for another 10 min at room temperature. Permeabilization and blocking were performed by adding blocking solution (5% BSA diluted in 1× SAPO) to one loading hole for 30 min and to the second loading hole for additional 30 min at room temperature. Afterward, the primary antibody was incubated overnight at 4°C (rat anti–α-tubulin, 2 μg/ml; MA1-80017, Invitrogen; diluted in 1× SAPO). The next day, samples were washed with PBS and stained with the secondary antibody (goat anti-rat Alexa Fluor Plus 647, 4 μg/ml; A48265, Invitrogen; diluted in 1× SAPO) and DAPI (1:1000; Thermo Fisher Scientific) added to one loading hole for 30 min and to the second loading hole for another 30 min at room temperature. Afterward, washing and mounting were performed as described above.

### Transcriptomics of migrating DCs

Bone marrow–derived DCs were incorporated into 3D collagen gels as described above. Collagen gels of different densities were obtained by using different bovine collagen stock dilutions to final concentrations of 1.7, 2.6, 3.5, and 4.4 mg/ml. Controls were performed by placing DCs into the same migration chambers but without collagen. Five hours after introduction of the CCL19 chemokine gradient, the gel was isolated from the chamber and immediately bathed in TRIzol. Subsequently, RNA extraction was performed following the “Total RNA extraction” protocol of the “Immunological Genome Project” (ImmGen.org). The amount of total RNA was quantified using the Qubit 2.0 Fluorometric Quantitation system (Thermo Fisher Scientific, Waltham, MA, USA), and the RNA integrity number was determined using the Experion Automated Electrophoresis System (Bio-Rad, Hercules, CA, USA). RNA-sequencing (RNA-seq) libraries were prepared with the TruSeq Stranded mRNA LT sample preparation kit (Illumina, San Diego, CA, USA) using Sciclone and Zephyr liquid handling workstations (PerkinElmer, Waltham, MA, USA) for pre- and post-PCR steps, respectively. Library concentrations were quantified with the Qubit 2.0 Fluorometric Quantitation system (Life Technologies, Carlsbad, CA, USA), and the size distribution was assessed using the Experion Automated Electrophoresis System (Bio-Rad, Hercules, CA, USA). Before sequencing on an Illumina HiSeq 2000 instrument following a 50–base pair single-end protocol, samples were diluted and pooled into NGS libraries in equimolar amounts. For analysis, sequencing reads were aligned to the mouse reference genome (version GRCm38.95) with STAR (version 2.7.0f). Expression values (TPM) were calculated with RSEM (version 1.3.0). Post-processing was performed in R/bioconductor (version 3.5.3) using default parameters if not indicated otherwise. Differential gene expression analysis was performed with “DEseq2” (version 1.22.2). An adjusted *P* value [false discovery rate (FDR)] of less than 0.1 was used to classify significantly changed expression.

### Phosphoproteomics of migrating DCs

Collagen migration assays were performed as described above. To achieve sufficient cell numbers allowing phosphoproteomic analysis, the experimental setup was adjusted to 1 × 10^7^ bone marrow–derived DCs per custom-made chamber (65 cm^2^). Collagen gels of different densities were obtained by using different bovine collagen stock solutions (PureCol and FibriCol, Advanced BioMatrix) to final concentrations of 1.7 and 5.6 mg/ml, respectively. Cells were incubated at 37°C and 5% CO_2_ for 90 min to enable the establishment of a CCL19 gradient and the initiation of cell migration. Afterward, the gel was isolated from the chamber, placed into a 15-ml Falcon tube, and immediately snap frozen in liquid nitrogen. After 15 min, the samples were transferred to −80°C. For phosphopeptide enrichment, the EasyPhos protocol (*67*) was adapted to our experimental settings. In short, cell and collagen were lysed in 4% sodium deoxycholate (SDC) lysis buffer and immediately heat-treated at 95°C. After sonification, samples were reduced with 10 mM tris[2-carboxy(ethyl)phosphine], alkylated with 40 mM 2-chloroacetamide, and digested for 18 hours with trypsin and lysC (1:100, w:w). Following phosphopeptide enrichment and depletion of the non-phosphorylated collagen, the digested peptides were desalted using styrene-divinylbenzene, reversed-phase sulfonate (SDB-RPS) StageTips. Desalted peptides (500 ng) were resolubilized in 6 μl of 2% acetonitrile (ACN) and 0.3% trifluoroacetic acid, injected into an Orbitrap Exploris 480 mass spectrometer (Thermo Fisher Scientific) coupled with an EASY-nLC 1000 system (Thermo Fisher Scientific), and analyzed in data-independent acquisition (DIA) mode. Peptides were separated at a flow rate of 300 nl/min with a 70-min gradient starting at 3% buffer B (80% ACN and 0.1% formic acid) and ramping to 19% in 40 min, 41% in 20 min, 90% in 5 min, and 95% in 5 min. The DIA MS data were acquired with the following parameters: scan range, 300 to 1650 mass/charge ratio (*m*/*z*), MS resolution, 60,000 at 200 *m*/*z*; and AGC target, 3 × 10^6^. The DIA MS/MS scan was performed in the HCD mode with the following parameters: 32 isolation windows with a resolution of 30,000; maximum injection time, 54 ms; stepped collision energy, 25, 27.5, and 30%; and AGC target, 3 × 10^6^. For DIA data analysis, MS raw files were processed using Spectronaut version 14 (Biognosys) using standard settings (*68*). The mouse UniProt FASTA database (April 2021) was used. The FDR was set to 1% at the protein and peptide precursor level. Further statistical analysis was performed with Perseus (1.6.1.1) and R (4.1.0).

### Imaging

#### Wide-field microscopy

Live-cell imaging was performed at 37°C, and supplementation with 5% CO_2_ in a humidified chamber if needed. Cell migration was recorded using conventional inverted wide-field DMi8 microscopes (Leica) using HC PL FLUOTAR 4×/0.5 PH0 air, HC PL FLUOTAR L 20×/0.40 PH1 air, HC PL APO 40×/0.9 PH3 air, and HC PL APO 100×/1.40 oil objectives, equipped with a Lumencor or pE-4000 light source (395, 475, 555, and 635 nm) and an incubation chamber, heated stage, and CO_2_ mixer (Pecon). For evaluation of microtubule nucleation dynamics, EB3-mCherry–expressing DCs were imaged at 5-s intervals. Acquisition of immunofluorescence samples was performed on an inverted wide-field DMi8 microscope (Leica) equipped with an HC PL APO 100×/1.47 oil objective.

#### Whole-mount ear skin imaging

Confocal microscopy of ear sheets was performed on a motorized stage at room temperature with an inverted microscope, equipped with an Airyscan module, a Plan-Apochromat 20×/0.8 objective, 488- and 561-nm laser lines, and a photomultiplier tube (PMT; all Zeiss). For all experiments, imaging software ZEN Black 2.3 SP1 was deployed.

#### Fluorescence recovery after photobleaching

For FRAP experiments, Jurkat T cells were electroporated with the indicated fluorescent reporter construct and loaded into an under-agarose migration assay without chemotactic stimulus as described above. FRAP experiments were performed at the Core Facility Bioimaging of the Biomedical Center with an inverted Leica TCS SP8X STED 3× equipped with two PMTs and three hybrid detectors (HyDs), Argon laser, WLL2 laser (470 to 670 nm), acousto-optical beam splitter, and 592- and 660-nm continuous-wave and 775-nm pulsed depletion lasers. Cells were recorded at 37°C and 5% CO_2_. Images were acquired in unidirectional scanning mode with an HC PL APO CS2 40×/1.30 oil objective, with an image pixel size of 142.6 nm and a pixel dwell time of 4.8 μs. The following fluorescence settings were used: GFP [excitation, 488 nm (Argon laser); and emission, 500 to 570 nm] or RFP (excitation, 514 nm; and emission, 536 to 661 nm), both being recorded with conventional PMTs. Five pre-bleach frames at a frame rate of 1 s were recorded, followed by bleaching at full laser power in zoom-in mode for two times. Initial signal recovery was recorded for 10 frames at a frame rate of 1 s, followed by extended post-bleach recording in 5-s time intervals for at least 2 min.

#### Stimulated emission depletion

STED microscopy was performed at the Core Facility Bioimaging of the Biomedical Center with an inverted Leica TCS SP8X STED 3× equipped with two PMTs and three HyDs, Argon laser, WLL2 laser (470 to 670 nm), acousto-optical beam splitter, and 592- and 660-nm continuous-wave and 775-nm pulsed depletion lasers. Images were acquired in unidirectional scanning mode with an HC PL APO CS2 100×/1.40 oil STED white objective, with an image pixel size of 25 nm and an accumulated pixel dwell time of 29.3 μs. For conventional confocal imaging, the following fluorescence settings were used: GFP (WLL2 pulsed excitation, 489 nm; and emission, 500 to 570 nm; PMT). For STED microscopy, the following settings were used: Alexa Fluor 594 (WLL2 pulsed excitation, 590 nm; and emission, 600 to 670 nm; depletion in 2D mode with a 775-nm pulsed laser). The signal was recorded with a hybrid detector in photon counting mode with time gating set to 0.5 to 6 ns and the corresponding reference wavelength of 590 nm. The recording was sequentially to avoid bleed-through.

### Image analysis

Fiji/ImageJ (*69*) and Imaris (Bitplane) were used for image processing. In general, only single, noninteracting cells were included for analysis to avoid effects of neighboring cells on cell path, cell speed, and centriolar dynamics.

Velocity of migrating DCs and Jurkat T cells along unidirectional paths (narrow straight and wide straight micro-channels) was analyzed using the tracking function of Imaris v9.7.2. For DC migration, cell nuclei were tracked with the following settings: object diameter, 12 μm; manually adjusted quality threshold; autoregressive motion tracking algorithm (maximum distance, 25 μm; gap size, 3); and minimum track duration, 15 min. For Jurkat T cell migration the following settings were applied: object diameter, 15 μm; manually adjusted quality threshold; autoregressive motion tracking algorithm (maximum distance, 15 μm; gap size, 3); and minimum track duration, 15 min. Pore translocation time and junction passing time, as well as centriolar distances, were manually quantified in Fiji.

Centrosome splitting (fracturing) was classified as a distance above 1.5 μm between the two individual centrioles in a centriolar pair. For more detailed characterization of centrosome fracturing in DCs, the distance was further categorized as close (0 to 1.49 μm), near (1.5 to 3.49 μm), short-distance separated (3.5 to 4.99 μm), and long-distance separated (above 5 μm). For 3T3 fibroblasts, centriolar distance was categorized as close (0 to 1.49 μm), near (1.5 to 2.99 μm), and far (above 3 μm). For characterization of fractured centrosomes by immunostainings, recorded cells were analyzed by categorizing fluorescence signal patterns as indicated. Dyrk3 localization was evaluated by measuring fluorescence intensity profiles along a 5-μm-long line determined by the centriolar axis using the Plot profile function in Fiji. Then, fluorescence values were normalized to the mean intensity value of the first and last four measured values, respectively. The competing protrusion length was measured using ImageJ by determining the maximal length for each protrusion during a productive path decision. The longest of those protrusions was defined as the main competing protrusion.

Characterization of intact centrosomes was performed by measuring mean fluorescence intensities of two circular regions of interest (radius, 0.35 μm) each centered to one centriole in summed *Z*-projections. Next, the ratio between both measured values was calculated choosing the higher value as divisor.

For analysis of microtubule nucleation rate and speed, cells that were well separated from other cells were randomly selected and cut out from raw movies. The EB3 comets within these cells were then tracked in Fiji using TrackMate v7.9.2 (*70*) (https://doi.org/10.1038/s41592-022-01507-1) with the following settings: LoG detector (object diameter, 0.65 μm), manually adjusted quality threshold, and minimum intensity filters (Kalman tracker: search radii, 7 and 10 μm; no frame gap). The resulting tracks were exported, and the presented statistics were derived with a custom MATLAB Script.

The fluorescence signal associated with the PCM was segmented using Ilastik’s pixel classifier workflow. In Fiji, movies related to a single experimental condition were combined, and the *y*-branching area of each channel was aligned using the “3D drift correct” plugin. Subsequently, the PCM was filtered on the basis of size, and shape analysis was conducted using the particle analyzer. The PCM was then skeletonized. Shape parameters, along with skeleton length, were extracted for further analysis.

DC migration in collagen matrices was analyzed using a custom-made cell tracking tool for ImageJ (*16*). In brief, cell migration image sequences were background corrected by subtracting the average of the entire sequence. Particle filtering was used to discard objects smaller or larger than the cells. Then, for each image in the sequence, the lateral displacement that optimizes its overlap with the previous frame was determined. Last, the migration velocity toward the chemokine source was calculated from the *y*-displacement and the time between two consecutive frames. To analyze Jurkat T cell migration, cells were manually tracked in Fiji/ImageJ using the manual tracking plugin. Migration speed, accumulated distance, and directionality were calculated using the ibidi chemotaxis and migration tool (*71*). The first 30 min of the recordings were excluded from analysis due to initial image drift. Cell shape analysis of DCs and Jurkat T cells was performed by manually outlining randomly selected cells after 200 min of recording to ensure a well-established chemokine gradient across the entire field of view. Afterward, cell shape descriptors were exported from ImageJ.

Distance analysis of lymph vessels in split ear sheets was performed using ImageJ. Maximum intensity *Z*-stack projections of confocal images from ear sheets were used to create binary images using Fiji. In binary images, lymphatic vessels were marked, and the distance from the vessel to every pixel, showing Cy3 fluorescence intensity above a specific threshold, was measured. For distance measurement, an in-house–generated MATLAB Script was used as described previously (*12*).

FRAP analysis was performed using ImageJ. The recovery rate was calculated by first subtracting background detector noise and then normalizing the mean fluorescence intensity of the bleached region to a non-bleached reference region inside the cell.

PH-Akt signal gradient was analyzed in DCs migrating under agarose toward a chemokine gradient consistently for at least 30 min. Cells were imaged every 5 min, and results were averaged over 6 to 20 time points for each cell. Fluorescence intensities of all pixels were extracted with a custom-made ImageJ script from images of cells segmented on the basis of threshold and manual input. Cells with centrioles 1.5 μm apart for at least two time points or more were considered to have a fractured centrosome. Using a custom-made R script (RStudio version 2022.12.0.353, R version 4.22, and the package tidyverse version 2.0.0) (*72*, *73*), the PH-Akt-dTomato to CETN2-GFP ratio was calculated from back to front of the cell: To normalize cells with different PH-Akt-dTomato and CETN2-GFP intensities, the respective fluorescence intensity was normalized to the intensity averaged over all time points of one cell, excluding a 1.5-μm area around each centriole for the entire analysis. Pixels were grouped in 50 (from 0 “back” to 1 “front”; see fig. S15D) or 2 (“back” and “front”; see fig. S15E) equally long segments, based on the distance to the rear of the cell in the direction of migration, normalized to the respective cell length. The ratio of the normalized PH-Akt to CETN2 signal was averaged for each of those segments, first in each time point and then over all time points for each cell.

### Statistics

All data that show individual cellular data points derive from cells from at least three independent biological replicates. All replicates were validated independently and pooled only when all showed similar results. Statistical analysis was conducted using GraphPad Prism using the appropriate tests according to normal or nonnormal data distribution: paired *t* test (Fig. 4, B and E), unpaired *t* test (Figs. 1, H and I; 2H; 3, D and G; 5, G to J; and 6, A to D; and figs. S2H; S4, B to E; S7D; S8, C and E; S9E; S10, B and D; S15B; and S17C), Fisher’s exact test (Fig. 6J and fig. S16C), Mann-Whitney (Figs. 1J; 2, D to F; 3, E, H, and J; 4, C and F; 5, B, C, and E; and 6, F to H and K; and figs. S14, A to C, and S15E), Kruskal-Wallis with Dunn’s multiple comparisons test (Fig. 1, E and F), two-way analysis of variance (ANOVA; fig. S3E), and linear regression fit (Fig. 6I). Error bars represent mean (fig. S2, E to G), means ± SD (Figs. 3J; 4, E and F; and 5, E and G to J; and figs. S8C, S10B, S15B, and S17C), means ± SEM (Figs. 1, H to J; 2, A, B, and H; 3, D, E, G, and H; 4, B and C; and 6, A to D; and figs. S2H, S8E, and S9E), means ± 95% confidence interval (CI; Figs. 2J and 4, B and D; and figs. S3E; S4, B to E; S5, B and C; S10D; and S15, D and E), and median ± 95% CI (Figs. 1, E and F; 2, D to F; 5, B and C; and 6, F to H and K; and figs. S2D; S7D; S9C; and S14, A to C).

## References

[1] V. Vogel, M. Sheetz, Local force and geometry sensing regulate cell functions. Nat. Rev. Mol. Cell Biol. 7, 265–275 (2006). 16607289 10.1038/nrm1890

[2] M. Raab, M. Gentili, H. de Belly, H. R. Thiam, P. Vargas, A. J. Jimenez, F. Lautenschlaeger, R. Voituriez, A. M. Lennon-Duménil, N. Manel, M. Piel, ESCRT III repairs nuclear envelope ruptures during cell migration to limit DNA damage and cell death. Science 352, 359–362 (2016). 27013426 10.1126/science.aad7611

[3] C. M. Denais, R. M. Gilbert, P. Isermann, A. L. McGregor, M. te Lindert, B. Weigelin, P. M. Davidson, P. Friedl, K. Wolf, J. Lammerding, Nuclear envelope rupture and repair during cancer cell migration. Science 352, 353–358 (2016). 27013428 10.1126/science.aad7297 PMC4833568

[4] A. Akhmanova, L. C. Kapitein, Mechanisms of microtubule organization in differentiated animal cells. Nat. Rev. Mol. Cell Biol. 23, 541–558 (2022). 35383336 10.1038/s41580-022-00473-y

[5] P. Robison, M. A. Caporizzo, H. Ahmadzadeh, A. I. Bogush, C. Y. Chen, K. B. Margulies, V. B. Shenoy, B. L. Prosser, Detyrosinated microtubules buckle and bear load in contracting cardiomyocytes. Science 352, aaf0659 (2016). 27102488 10.1126/science.aaf0659 PMC5441927

[6] L. Schaedel, K. John, J. Gaillard, M. V. Nachury, L. Blanchoin, M. Théry, Microtubules self-repair in response to mechanical stress. Nat. Mater. 14, 1156–1163 (2015). 26343914 10.1038/nmat4396 PMC4620915

[7] M. Bornens, The centrosome in cells and organisms. Science 335, 422–426 (2012). 22282802 10.1126/science.1209037

[8] M. Panic, S. Hata, A. Neuner, E. Schiebel, The centrosomal linker and microtubules provide dual levels of spatial coordination of centrosomes. PLOS Genet. 11, e1005243 (2015). 26001056 10.1371/journal.pgen.1005243 PMC4441491

[9] K. M. Yamada, M. Sixt, Mechanisms of 3D cell migration. Nat. Rev. Mol. Cell Biol. 20, 738–752 (2019). 31582855 10.1038/s41580-019-0172-9

[10] T. Worbs, S. I. Hammerschmidt, R. Förster, Dendritic cell migration in health and disease. Nat. Rev. Immunol. 17, 30–48 (2017). 27890914 10.1038/nri.2016.116

[11] A.-K. Weier, M. Homrich, S. Ebbinghaus, P. Juda, E. Miková, R. Hauschild, L. Zhang, T. Quast, E. Mass, A. Schlitzer, W. Kolanus, S. Burgdorf, O. J. Gruß, M. Hons, S. Wieser, E. Kiermaier, Multiple centrosomes enhance migration and immune cell effector functions of mature dendritic cells. J. Cell Biol. 221, e202107134 (2022). 36214847 10.1083/jcb.202107134 PMC9555069

[12] A. Kopf, J. Renkawitz, R. Hauschild, I. Girkontaite, K. Tedford, J. Merrin, O. Thorn-Seshold, D. Trauner, H. Häcker, K.-D. Fischer, E. Kiermaier, M. Sixt, Microtubules control cellular shape and coherence in amoeboid migrating cells. J. Cell Biol. 219, e201907154 (2020). 32379884 10.1083/jcb.201907154 PMC7265309

[13] H. Higginbotham, S. Bielas, T. Tanaka, J. G. Gleeson, Transgenic mouse line with green-fluorescent protein-labeled Centrin 2 allows visualization of the centrosome in living cells. Transgenic Res. 13, 155–164 (2004). 15198203 10.1023/b:trag.0000026071.41735.8e

[14] H. Dang, E. Schiebel, Emerging roles of centrosome cohesion. Open Biol. 12, 220229 (2022). 36285440 10.1098/rsob.220229 PMC9597181

[15] M. Schliwa, K. B. Pryzwansky, U. Euteneuer, Centrosome splitting in neutrophils: An unusual phenomenon related to cell activation and motility. Cell 31, 705–717 (1982). 7159931 10.1016/0092-8674(82)90325-7

[16] A. Leithner, A. Eichner, J. Müller, A. Reversat, M. Brown, J. Schwarz, J. Merrin, D. J. J. de Gorter, F. Schur, J. Bayerl, I. de Vries, S. Wieser, R. Hauschild, F. P. L. Lai, M. Moser, D. Kerjaschki, K. Rottner, J. V. Small, T. E. B. Stradal, M. Sixt, Diversified actin protrusions promote environmental exploration but are dispensable for locomotion of leukocytes. Nat. Cell Biol. 18, 1253–1259 (2016). 27775702 10.1038/ncb3426

[17] L. K. Fritz-Laylin, M. Riel-Mehan, B.-C. Chen, S. J. Lord, T. D. Goddard, T. E. Ferrin, S. M. Nicholson-Dykstra, H. Higgs, G. T. Johnson, E. Betzig, R. D. Mullins, Actin-based protrusions of migrating neutrophils are intrinsically lamellar and facilitate direction changes. eLife 6, e26990 (2017). 28948912 10.7554/eLife.26990 PMC5614560

[18] J. Kroll, R. Hauschild, A. Kuznetcov, K. Stefanowski, M. D. Hermann, J. Merrin, L. Shafeek, A. Müller-Taubenberger, J. Renkawitz, Adaptive pathfinding by nucleokinesis during amoeboid migration. EMBO J. 42, e114557 (2023). 37987147 10.15252/embj.2023114557 PMC10711653

[19] J. B. Woodruff, B. F. Gomes, P. O. Widlund, J. Mahamid, A. Honigmann, A. A. Hyman, The centrosome is a selective condensate that nucleates microtubules by concentrating tubulin. Cell 169, 1066–1077.e10 (2017). 28575670 10.1016/j.cell.2017.05.028

[20] J. W. Raff, Phase separation and the centrosome: A fait accompli? Trends Cell Biol. 29, 612–622 (2019). 31076235 10.1016/j.tcb.2019.04.001

[21] Y. Shin, C. P. Brangwynne, Liquid phase condensation in cell physiology and disease. Science 357, eaaf4382 (2017). 28935776 10.1126/science.aaf4382

[22] F. Wippich, B. Bodenmiller, M. G. Trajkovska, S. Wanka, R. Aebersold, L. Pelkmans, Dual specificity kinase DYRK3 couples stress granule condensation/dissolution to mTORC1 signaling. Cell 152, 791–805 (2013). 23415227 10.1016/j.cell.2013.01.033

[23] A. K. Rai, J.-X. Chen, M. Selbach, L. Pelkmans, Kinase-controlled phase transition of membraneless organelles in mitosis. Nature 559, 211–216 (2018). 29973724 10.1038/s41586-018-0279-8

[24] J. Renkawitz, A. Kopf, J. Stopp, I. de Vries, M. K. Driscoll, J. Merrin, R. Hauschild, E. S. Welf, G. Danuser, R. Fiolka, M. Sixt, Nuclear positioning facilitates amoeboid migration along the path of least resistance. Nature 568, 546–550 (2019). 30944468 10.1038/s41586-019-1087-5 PMC7217284

[25] P. Kameritsch, J. Renkawitz, Principles of leukocyte migration strategies. Trends Cell Biol. 30, 818–832 (2020). 32690238 10.1016/j.tcb.2020.06.007

[26] A. Dammermann, A. Merdes, Assembly of centrosomal proteins and microtubule organization depends on PCM-1. J. Cell Biol. 159, 255–266 (2002). 12403812 10.1083/jcb.200204023 PMC2173044

[27] E. J. Lawrence, M. Zanic, L. M. Rice, CLASPs at a glance. J. Cell Sci. 133, jcs243097 (2020). 32332092 10.1242/jcs.243097 PMC7188440

[28] G. C. Ortega, S. Falk, P. A. Johansson, E. Peyre, L. Broix, S. K. Sahu, W. Hirst, T. Schlichthaerle, C. De Juan Romero, K. Draganova, S. Vinopal, K. Chinnappa, A. Gavranovic, T. Karakaya, T. Steininger, J. Merl-Pham, R. Feederle, W. Shao, S.-H. Shi, S. M. Hauck, R. Jungmann, F. Bradke, V. Borrell, A. Geerlof, S. Reber, V. K. Tiwari, W. B. Huttner, M. Wilsch-Bräuninger, L. Nguyen, M. Götz, The centrosome protein AKNA regulates neurogenesis via microtubule organization. Nature 567, 113–117 (2019). 30787442 10.1038/s41586-019-0962-4

[29] S. Croessmann, H. Y. Wong, D. J. Zabransky, D. Chu, J. Mendonca, A. Sharma, M. Mohseni, D. M. Rosen, R. B. Scharpf, J. Cidado, R. L. Cochran, H. A. Parsons, W. B. Dalton, B. Erlanger, B. Button, K. Cravero, K. Kyker-Snowman, J. A. Beaver, S. Kachhap, P. J. Hurley, J. Lauring, B. H. Park, NDRG1 links p53 with proliferation-mediated centrosome homeostasis and genome stability. Proc. Natl. Acad. Sci. U.S.A. 112, 11583–11588 (2015). 26324937 10.1073/pnas.1503683112 PMC4577188

[30] N. H. Cho, K. C. Cheveralls, A.-D. Brunner, K. Kim, A. C. Michaelis, P. Raghavan, H. Kobayashi, L. Savy, J. Y. Li, H. Canaj, J. Y. S. Kim, E. M. Stewart, C. Gnann, F. M. Carthy, J. P. Cabrera, R. M. Brunetti, B. B. Chhun, G. Dingle, M. Y. Hein, B. Huang, S. B. Mehta, J. S. Weissman, R. Gómez-Sjöberg, D. N. Itzhak, L. A. Royer, M. Mann, M. D. Leonetti, OpenCell: Endogenous tagging for the cartography of human cellular organization. Science 375, eabi6983 (2022). 35271311 10.1126/science.abi6983 PMC9119736

[31] P. J. Thul, L. Åkesson, M. Wiking, D. Mahdessian, A. Geladaki, H. A. Blal, T. Alm, A. Asplund, L. Björk, L. M. Breckels, A. Bäckström, F. Danielsson, L. Fagerberg, J. Fall, L. Gatto, C. Gnann, S. Hober, M. Hjelmare, F. Johansson, S. Lee, C. Lindskog, J. Mulder, C. M. Mulvey, P. Nilsson, P. Oksvold, J. Rockberg, R. Schutten, J. M. Schwenk, Å. Sivertsson, E. Sjöstedt, M. Skogs, C. Stadler, D. P. Sullivan, H. Tegel, C. Winsnes, C. Zhang, M. Zwahlen, A. Mardinoglu, F. Pontén, K. von Feilitzen, K. S. Lilley, M. Uhlén, E. Lundberg, A subcellular map of the human proteome. Science 356, eaal3321 (2017). 28495876 10.1126/science.aal3321

[32] H. Cheng, Y.-L. Kao, T. Chen, L. Sharma, W.-T. Yang, Y.-C. Chuang, S.-H. Huang, H.-R. Lin, Y.-S. Huang, C.-L. Kao, L.-W. Yang, R. Bearon, H.-C. Cheng, K.-C. Hsia, Y.-C. Lin, Actin filaments form a size-dependent diffusion barrier around centrosomes. EMBO Rep. 24, e54935 (2023). 36314725 10.15252/embr.202254935 PMC9827556

[33] A. M. Fry, T. Mayor, P. Meraldi, Y.-D. Stierhof, K. Tanaka, E. A. Nigg, C-Nap1, a novel centrosomal coiled-coil protein and candidate substrate of the cell cycle-regulated protein kinase Nek2. J. Cell Biol. 141, 1563–1574 (1998). 9647649 10.1083/jcb.141.7.1563 PMC2133000

[34] F. Gaertner, P. Reis-Rodrigues, I. de Vries, M. Hons, J. Aguilera, M. Riedl, A. Leithner, S. Tasciyan, A. Kopf, J. Merrin, V. Zheden, W. A. Kaufmann, R. Hauschild, M. Sixt, WASp triggers mechanosensitive actin patches to facilitate immune cell migration in dense tissues. Dev. Cell 57, 47–62.e9 (2022). 34919802 10.1016/j.devcel.2021.11.024 PMC8751638

[35] D. Inoue, D. Obino, J. Pineau, F. Farina, J. Gaillard, C. Guerin, L. Blanchoin, A.-M. Lennon-Duménil, M. Théry, Actin filaments regulate microtubule growth at the centrosome. EMBO J. 38, e99630 (2019). 30902847 10.15252/embj.201899630 PMC6545561

[36] F. Farina, J. Gaillard, C. Guérin, Y. Couté, J. Sillibourne, L. Blanchoin, M. Théry, The centrosome is an actin-organizing centre. Nat. Cell Biol. 18, 65–75 (2016). 26655833 10.1038/ncb3285 PMC4880044

[37] E. Vitiello, P. Moreau, V. Nunes, A. Mettouchi, H. Maiato, J. G. Ferreira, I. Wang, M. Balland, Acto-myosin force organization modulates centriole separation and PLK4 recruitment to ensure centriole fidelity. Nat. Commun. 10, 52 (2019). 30604763 10.1038/s41467-018-07965-6 PMC6318293

[38] R. Basto, J. Lau, T. Vinogradova, A. Gardiol, C. G. Woods, A. Khodjakov, J. W. Raff, Flies without centrioles. Cell 125, 1375–1386 (2006). 16814722 10.1016/j.cell.2006.05.025

[39] A. Efimov, A. Kharitonov, N. Efimova, J. Loncarek, P. M. Miller, N. Andreyeva, P. Gleeson, N. Galjart, A. R. R. Maia, I. X. McLeod, J. R. Yates, H. Maiato, A. Khodjakov, A. Akhmanova, I. Kaverina, Asymmetric CLASP-dependent nucleation of noncentrosomal microtubules at the trans-golgi network. Dev. Cell 12, 917–930 (2007). 17543864 10.1016/j.devcel.2007.04.002 PMC2705290

[40] L. C. Klemm, R. A. Denu, L. E. Hind, B. L. Rocha-Gregg, M. E. Burkard, A. Huttenlocher, Centriole and Golgi microtubule nucleation are dispensable for the migration of human neutrophil-like cells. Mol. Biol. Cell 32, 1545–1556 (2021). 34191538 10.1091/mbc.E21-02-0060 PMC8351748

[41] M. Martin, A. Veloso, J. Wu, E. A. Katrukha, A. Akhmanova, Control of endothelial cell polarity and sprouting angiogenesis by non-centrosomal microtubules. eLife 7, e33864 (2018). 29547120 10.7554/eLife.33864 PMC5898915

[42] M. Piel, P. Meyer, A. Khodjakov, C. L. Rieder, M. Bornens, The respective contributions of the mother and daughter centrioles to centrosome activity and behavior in vertebrate cells. J. Cell Biol. 149, 317–330 (2000). 10769025 10.1083/jcb.149.2.317 PMC2175166

[43] B. P. Bouchet, A. Akhmanova, Microtubules in 3D cell motility. J. Cell Sci. 130, 39–50 (2017). 28043967 10.1242/jcs.189431

[44] P. Várnai, T. Balla, Visualization of phosphoinositides that bind pleckstrin homology domains: Calcium- and agonist-induced dynamic changes and relationship to myo-[3H]inositol-labeled phosphoinositide pools. J. Cell Biol. 143, 501–510 (1998). 9786958 10.1083/jcb.143.2.501 PMC2132833

[45] I. Stötzel, A.-K. Weier, A. Sarkar, S. Som, P. Konopka, E. Miková, J. Böthling, M. Homrich, L. Schaedel, U. Kazmaier, K. Symeonidis, Z. Abdullah, S. Uderhardt, M. Hons, R. Paul, H. Rieger, E. Kiermaier, Multiple clustered centrosomes in antigen-presenting cells foster T cell activation without MTOC polarization. bioRxiv 604057 (2024). 10.1101/2024.07.18.604057.

[46] Y. L. Wong, J. V. Anzola, R. L. Davis, M. Yoon, A. Motamedi, A. Kroll, C. P. Seo, J. E. Hsia, S. K. Kim, J. W. Mitchell, B. J. Mitchell, A. Desai, T. C. Gahman, A. K. Shiau, K. Oegema, Reversible centriole depletion with an inhibitor of Polo-like kinase 4. Science 348, 1155–1160 (2015). 25931445 10.1126/science.aaa5111 PMC4764081

[47] S. Alberti, A. A. Hyman, Biomolecular condensates at the nexus of cellular stress, protein aggregation disease and ageing. Nat. Rev. Mol. Cell Biol. 22, 196–213 (2021). 33510441 10.1038/s41580-020-00326-6

[48] S. Hata, A. P. Peidro, M. Panic, P. Liu, E. Atorino, C. Funaya, U. Jäkle, G. Pereira, E. Schiebel, The balance between KIFC3 and EG5 tetrameric kinesins controls the onset of mitotic spindle assembly. Nat. Cell Biol. 21, 1138–1151 (2019). 31481795 10.1038/s41556-019-0382-6

[49] S. G. Pereira, M. A. D. Louro, M. Bettencourt-Dias, Biophysical and quantitative principles of centrosome biogenesis and structure. Annu. Rev. Cell Dev. Biol. 37, 43–63 (2021). 34314592 10.1146/annurev-cellbio-120219-051400

[50] J. Yang, M. Adamian, T. Li, Rootletin interacts with C-Nap1 and may function as a physical linker between the pair of centrioles/basal bodies in cells. Mol. Biol. Cell 17, 1033–1040 (2006). 16339073 10.1091/mbc.E05-10-0943 PMC1356609

[51] H. Dang, A. Martin-Villalba, E. Schiebel, Centrosome linker protein C-Nap1 maintains stem cells in mouse testes. EMBO Rep. 23, e53805 (2022). 35599622 10.15252/embr.202153805 PMC9253759

[52] J. Kroll, J. Renkawitz, Principles of organelle positioning in motile and non-motile cells. EMBO Rep. 25, 2172–2187 (2024). 38627564 10.1038/s44319-024-00135-4 PMC11094012

[53] F. J. Calero-Cuenca, C. S. Janota, E. R. Gomes, Dealing with the nucleus during cell migration. Curr. Opin. Cell Biol. 50, 35–41 (2018). 29454272 10.1016/j.ceb.2018.01.014

[54] P. van Bergeijk, C. C. Hoogenraad, L. C. Kapitein, Right time, right place: Probing the functions of organelle positioning. Trends Cell Biol. 26, 121–134 (2016). 26541125 10.1016/j.tcb.2015.10.001

[55] S. Phuyal, P. Romani, S. Dupont, H. Farhan, Mechanobiology of organelles: Illuminating their roles in mechanosensing and mechanotransduction. Trends Cell Biol. 33, 1049–1061 (2023). 37236902 10.1016/j.tcb.2023.05.001

[56] Y. Kalukula, A. D. Stephens, J. Lammerding, S. Gabriele, Mechanics and functional consequences of nuclear deformations. Nat. Rev. Mol. Cell Biol. 23, 583–602 (2022). 35513718 10.1038/s41580-022-00480-z PMC9902167

[57] V. Redecke, R. Wu, J. Zhou, D. Finkelstein, V. Chaturvedi, A. A. High, H. Häcker, Hematopoietic progenitor cell lines with myeloid and lymphoid potential. Nat. Methods 10, 795–803 (2013). 23749299 10.1038/nmeth.2510 PMC4131762

[58] A. Leithner, J. Renkawitz, I. de Vries, R. Hauschild, H. Häcker, M. Sixt, Fast and efficient genetic engineering of hematopoietic precursor cells for the study of dendritic cell migration. Eur. J. Immunol. 48, 1074–1077 (2018). 29436709 10.1002/eji.201747358

[59] P. Datlinger, A. F. Rendeiro, C. Schmidl, T. Krausgruber, P. Traxler, J. Klughammer, L. C. Schuster, A. Kuchler, D. Alpar, C. Bock, Pooled CRISPR screening with single-cell transcriptome readout. Nat. Methods 14, 297–301 (2017). 28099430 10.1038/nmeth.4177 PMC5334791

[60] J. L. Schmid-Burgk, T. Schmidt, M. M. Gaidt, K. Pelka, E. Latz, T. S. Ebert, V. Hornung, OutKnocker: A web tool for rapid and simple genotyping of designer nuclease edited cell lines. Genome Res. 24, 1719–1723 (2014). 25186908 10.1101/gr.176701.114 PMC4199374

[61] M. L. Heuzé, G. H. N. S. Narayana, J. D’Alessandro, V. Cellerin, T. Dang, D. S. Williams, J. C. V. Hest, P. Marcq, R.-M. Mège, B. Ladoux, Myosin II isoforms play distinct roles in adherens junction biogenesis. eLife 8, e46599 (2019). 31486768 10.7554/eLife.46599 PMC6756789

[62] Y.-N. Lin, C.-T. Wu, Y.-C. Lin, W.-B. Hsu, C.-J. C. Tang, C.-W. Chang, T. K. Tang, CEP120 interacts with CPAP and positively regulates centriole elongation. J. Cell Biol. 202, 211–219 (2013). 23857771 10.1083/jcb.201212060 PMC3718976

[63] J. Hageman, H. H. Kampinga, Computational analysis of the human HSPH/HSPA/DNAJ family and cloning of a human HSPH/HSPA/DNAJ expression library. Cell Stress Chaperones 14, 1–21 (2009). 18686016 10.1007/s12192-008-0060-2 PMC2673897

[64] J. Kroll, M. J. A. Ruiz-Fernandez, M. B. Braun, J. Merrin, J. Renkawitz, Quantifying the probing and selection of microenvironmental pores by motile immune cells. Curr. Protoc. 2, e407 (2022). 35384410 10.1002/cpz1.407

[65] J. Renkawitz, K. Schumann, M. Weber, T. Lämmermann, H. Pflicke, M. Piel, J. Polleux, J. P. Spatz, M. Sixt, Adaptive force transmission in amoeboid cell migration. Nat. Cell Biol. 11, 1438–1443 (2009). 19915557 10.1038/ncb1992

[66] M. Sixt, T. Lämmermann, In vitro analysis of chemotactic leukocyte migration in 3D environments. Methods Mol. Biol. 769, 149–165 (2011). 21748675 10.1007/978-1-61779-207-6_11

[67] S. J. Humphrey, O. Karayel, D. E. James, M. Mann, High-throughput and high-sensitivity phosphoproteomics with the EasyPhos platform. Nat. Protoc. 13, 1897–1916 (2018). 30190555 10.1038/s41596-018-0014-9

[68] R. B. Kitata, W.-K. Choong, C.-F. Tsai, P.-Y. Lin, B.-S. Chen, Y.-C. Chang, A. I. Nesvizhskii, T.-Y. Sung, Y.-J. Chen, A data-independent acquisition-based global phosphoproteomics system enables deep profiling. Nat. Commun. 12, 2539 (2021). 33953186 10.1038/s41467-021-22759-z PMC8099862

[69] C. A. Schneider, W. S. Rasband, K. W. Eliceiri, NIH Image to ImageJ: 25 Years of image analysis. Nat. Methods 9, 671–675 (2012). 22930834 10.1038/nmeth.2089 PMC5554542

[70] J.-Y. Tinevez, N. Perry, J. Schindelin, G. M. Hoopes, G. D. Reynolds, E. Laplantine, S. Y. Bednarek, S. L. Shorte, K. W. Eliceiri, TrackMate: An open and extensible platform for single-particle tracking. Methods 115, 80–90 (2017). 27713081 10.1016/j.ymeth.2016.09.016

[71] R. Zantl, E. Horn, Chemotaxis of slow migrating mammalian cells analysed by video microscopy. Methods Mol. Biol. 769, 191–203 (2011). 21748677 10.1007/978-1-61779-207-6_13

[72] R Core Team, R: A language and environment for statistical computing (R Foundation for Statistical Computing, Vienna, Austria, 2022); www.R-project.org/.

[73] H. Wickham, M. Averick, J. Bryan, W. Chang, L. McGowan, R. François, G. Grolemund, A. Hayes, L. Henry, J. Hester, M. Kuhn, T. Pedersen, E. Miller, S. Bache, K. Müller, J. Ooms, D. Robinson, D. Seidel, V. Spinu, K. Takahashi, D. Vaughan, C. Wilke, K. Woo, H. Yutani, Welcome to the Tidyverse. J. Open Source Softw. 4, 1686 (2019).

