# Supplementary material for: Protecting centrosomes from fracturing enables efficient cell navigation
Source: Sci Adv. 2025 Apr 25;11(17):eadx4047. doi: 10.1126/sciadv.adx4047 (PMC12024656; doi:10.1126/sciadv.adx4047)
Supplement: Supplementary file 1 — Figs. S1 to S17 Legends for movies S1 to S10 [file sciadv.adx4047_sm.pdf]

Science Advances
AAAS

Supplementary Materials for

Protecting centrosomes from fracturing enables efficient cell navigation

Madeleine T. Schmitt *et al.*

Corresponding author: Jörg Renkawitz, joerg.renkawitz@med.uni-muenchen.de

Sci. Adv. **11**, eadx4047 (2025)
DOI: 10.1126/sciadv.adx4047

The PDF file includes:

Figs. S1 to S17
Legends for movies S1 to S10

Other Supplementary Material for this manuscript includes the following:

Movies S1 to S10

Fig. S1. Dynamics of the centriole pair during DC migration along unidirectional paths.

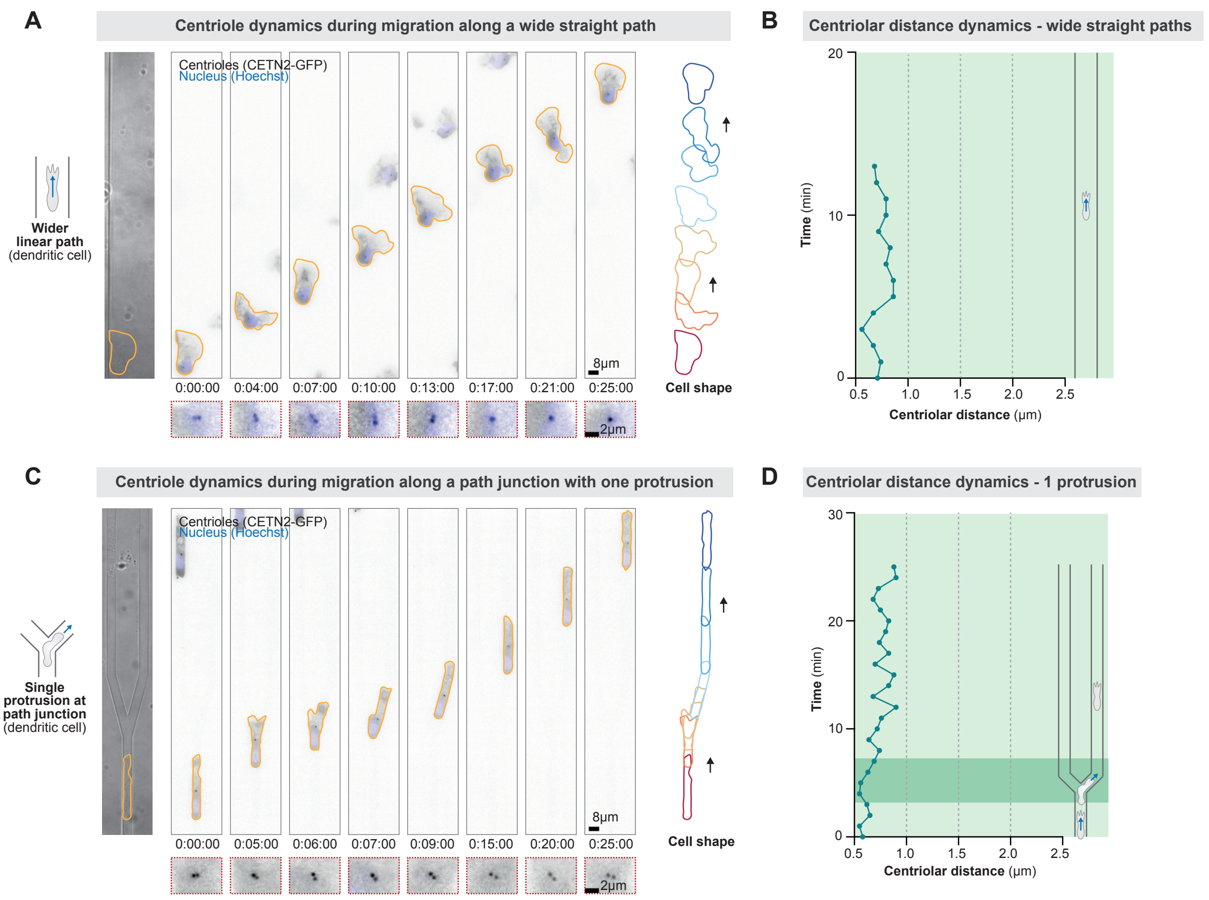

Fig. S1.

Dynamics of the centriole pair during DC migration along unidirectional paths. **(A)** Representative CETN2-GFP (centriole pair; black; enlargement in red dashed boxes) expressing dendritic cell (DC) stained with Hoechst (nucleus; blue) migrating along a unidirectional straight path (50 µm wide linear microchannel). **(B)** Centriolar distance dynamics during migration as depicted in (A); note the stable proximity of the individual centrioles. See Fig. 1E for quantification of centriolar distances during migration in this microenvironment composed of wide straight paths. **(C)** Representative CETN2-GFP (centriole pair; black; enlargement in red dashed boxes) expressing DC stained with Hoechst (nucleus; blue) migrating through a path junction (Y microchannel). Note that this specific cell has only one cell front at the path junction, and directly follows this cell protrusion into one of the two alternative paths. **(D)** Centriolar distance dynamics during migration as depicted in (C); note the stable proximity of the individual centrioles. See Fig. 1F for quantification of centriolar distances while cells explore the alternative paths with one protrusion (and in comparison, to two protrusions). All data show representative cells from at least three independent biological replicates. Time is indicated as h:min:s.

Fig. S2. Transcriptomics of migrating dendritic cells and pharmacological inhibition of Dyrk3 during dendritic cell and T cell migration, as well as expression of a dominant-negative Dyrk3 mutant in 3T3 fibroblasts.

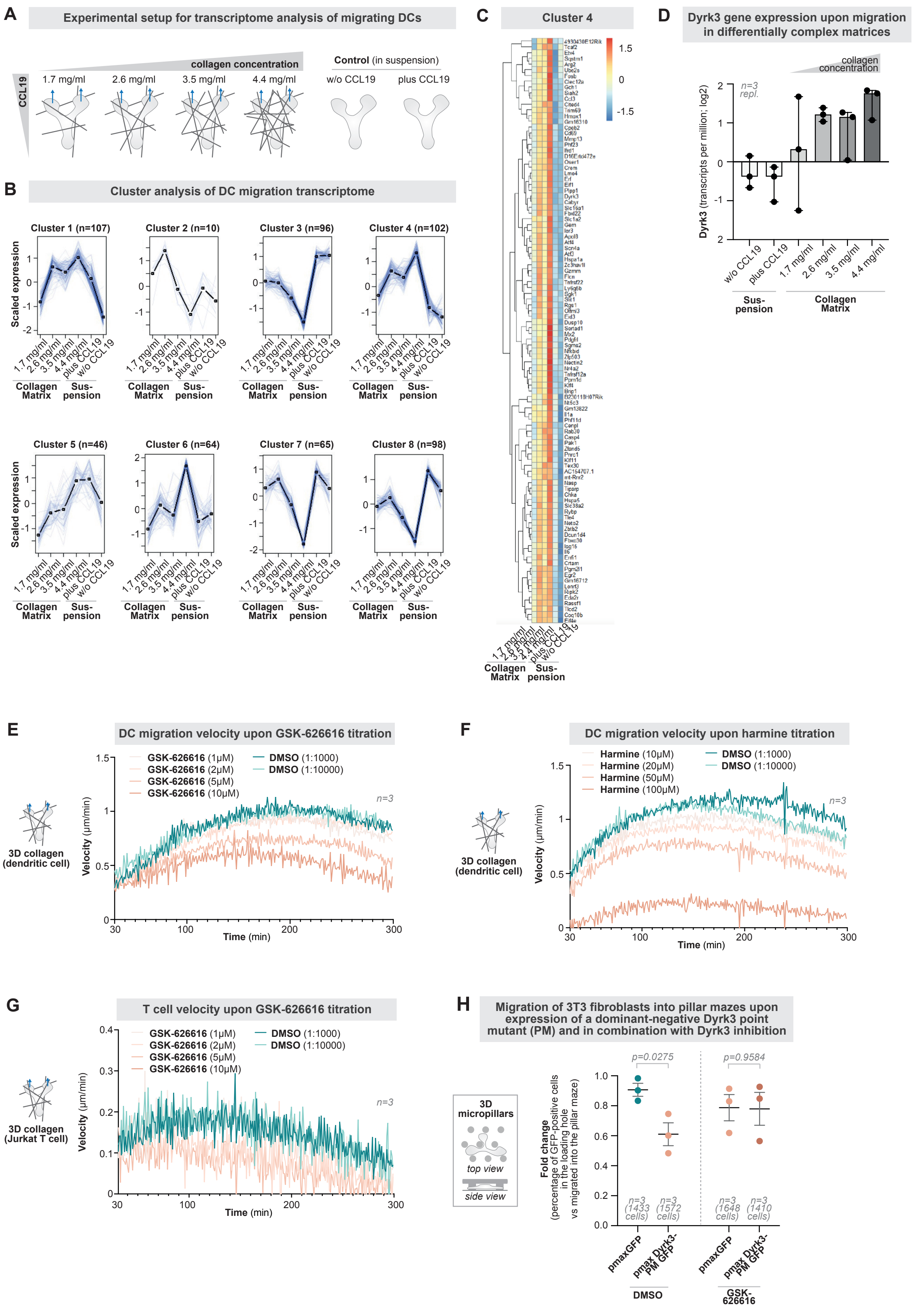

Fig. S2.

Transcriptomics of migrating dendritic cells (DCs) and pharmacological inhibition of Dyrk3 during DC and T cell migration, as well as expression of a dominant-negative Dyrk3 mutant in 3T3 fibroblasts. **(A)** Scheme of the experimental setup to investigate the transcriptome of migrating DCs in differentially complex collagen matrices (see 'Materials and Methods' for details). **(B)** Cluster analysis of differentially expressed genes derived from (A). **(C)** Close up view of genes in cluster 4, which upregulate their gene expression in more complex collagen matrices. **(D)** Differential gene expression of Dyrk3. **(E)** DC migration in three-dimensional (3D) collagen matrices (1.7 mg/ml) along a CCL19 chemokine gradient in the presence of different concentrations of GSK-626616 (Dyrk3 inhibitor) or DMSO (control). The dataset for 5 μM GSK-626616 is also shown in main Figure 2A. **(F)** DC migration in three-dimensional (3D) collagen matrices (1.7 mg/ml) along a CCL19 chemokine gradient in the presence of different concentrations of harmine (Dyrk inhibitor) or DMSO (control). The dataset for 50 μM harmine is also shown in main Figure 2B. **(G)** Jurkat T cell migration in three-dimensional (3D) collagen matrices (1.3 mg/ml) along a CXCL12 chemokine gradient in the presence of different concentrations of GSK-626616 (Dyrk3 inhibitor) or DMSO (control). **(H)** Fraction of GFP-positive 3T3 fibroblasts migrating into the pillar maze normalized to the fraction of GFP-positive 3T3 fibroblasts in the loading hole. Cells expressed a dominant-negative (DN) EGFP-Dyrk3 K218M mutant or the corresponding empty EGFP plasmid, either in the presence of GSK-626616 (Dyrk3 inhibitor) or DMSO (control). All data derive from at least three independent biological replicates.

Fig. S3. Fluorescence recovery after photobleaching (FRAP) of centrosomal proteins shows altered diffusion dynamics upon impairing Dyrk3 activity.

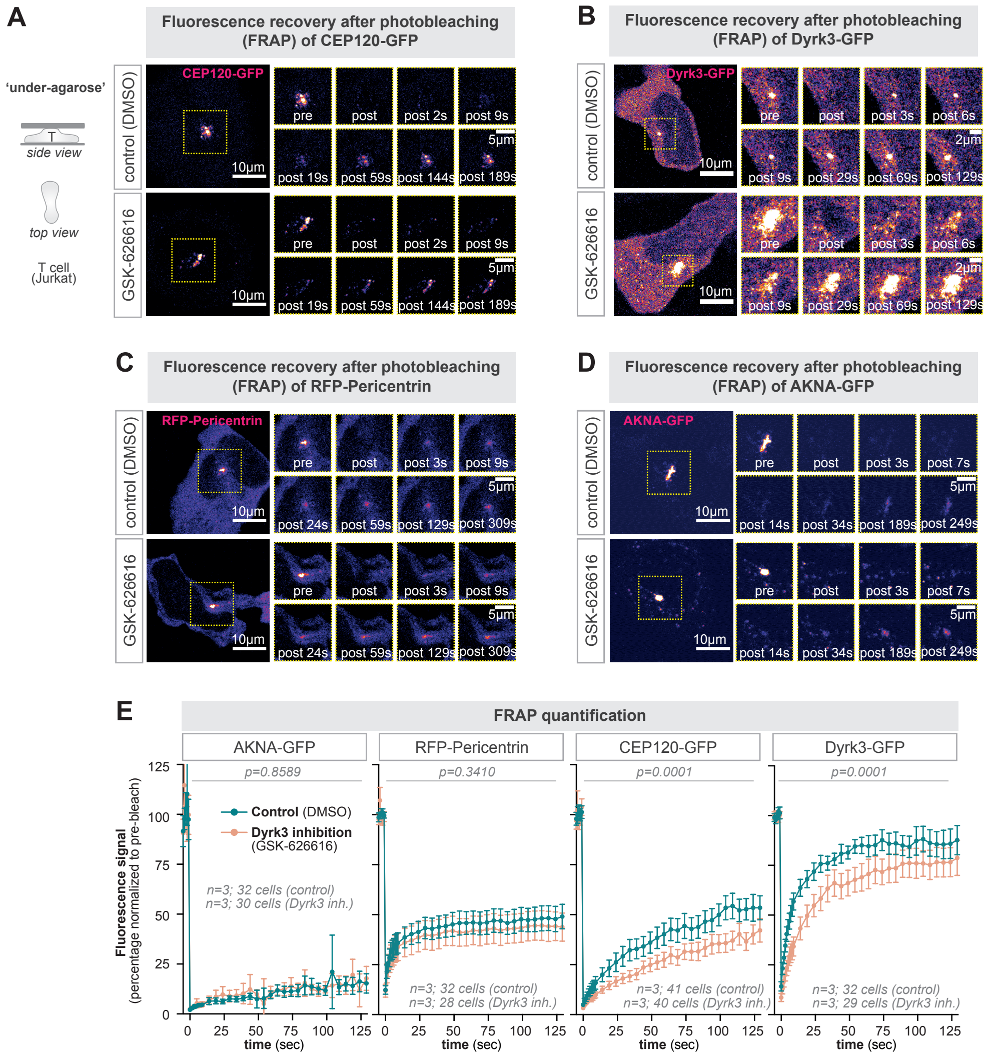

Fig. S3.

Fluorescence recovery after photobleaching (FRAP) of centrosomal proteins shows altered diffusion dynamics upon impairing Dyrk3 activity. **(A)** Representative CEP120-GFP (fire-color coded; enlargement in yellow dashed boxes) expressing Jurkat T cells before, immediately after, and for an extended time after bleaching of the CEP120 signal in the presence of 5 µM GSK-626616 or DMSO (control). **(B)** Representative Dyrk3-GFP (fire-color coded; enlargement in yellow dashed boxes) expressing Jurkat T cells before, immediately after, and for an extended time after bleaching of the Dyrk3 signal in the presence of 5 µM GSK-626616 or DMSO (control). **(C)** Representative RFP-Pericentrin (fire-color coded; enlargement in yellow dashed boxes) expressing Jurkat T cells before, immediately after, and for an extended time after bleaching of the Pericentrin signal in the presence of 5 µM GSK-626616 or DMSO (control). **(D)** Representative AKNA-GFP (fire-color coded; enlargement in yellow dashed boxes) expressing Jurkat T cells before, immediately after, and for an extended time after bleaching of the AKNA signal in the presence of 5 µM GSK-626616 or DMSO (control). **(E)** Quantification of fluorescence signal recovery after photobleaching as shown in (A-D). All data derive from at least three independent biological replicates.

Fig. S4. Centrosome deformation dynamics and fracturing frequency during migration along unidirectional paths, in microenvironmental confinement, and in confining mazes.

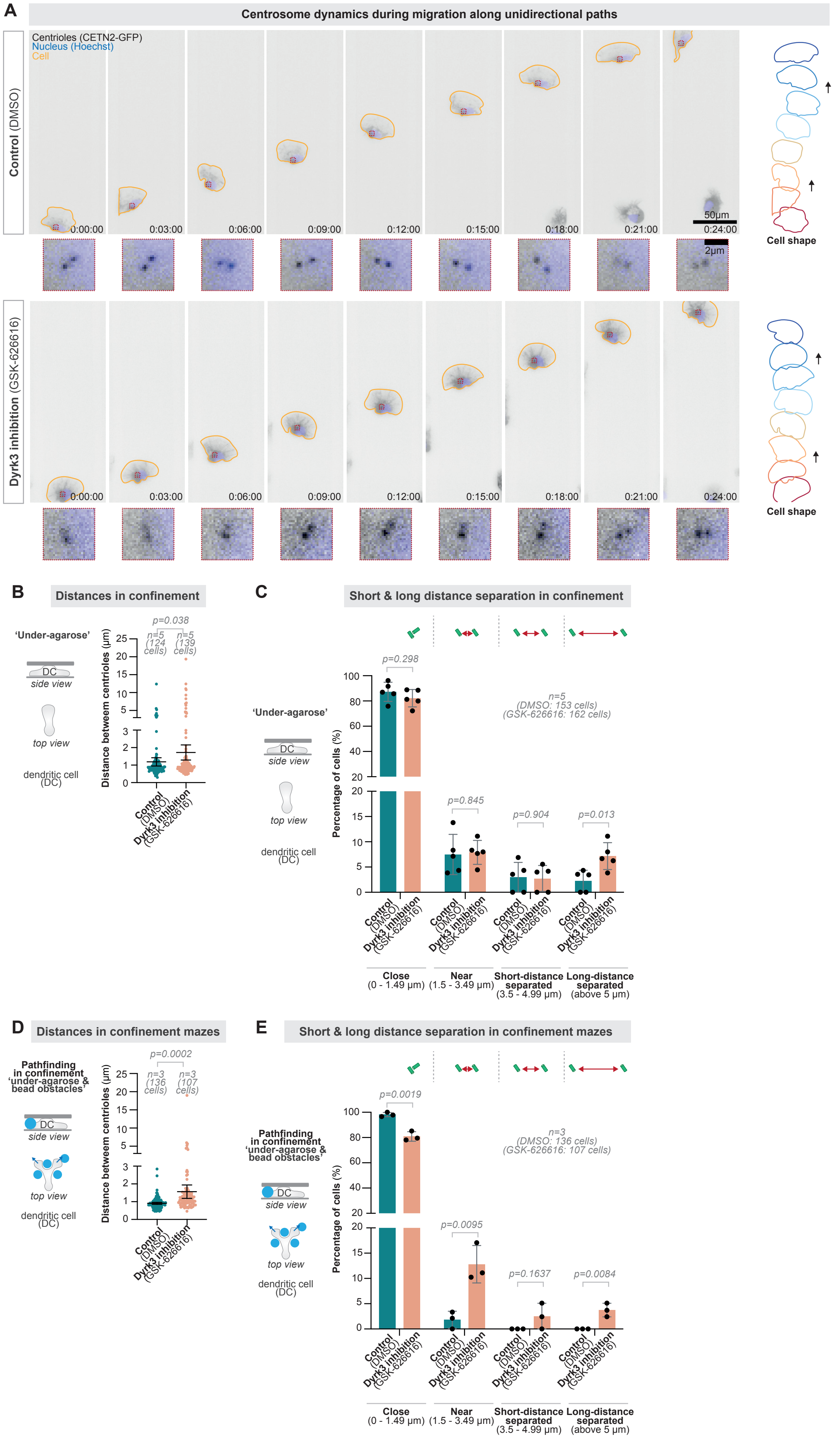

Fig. S4.

Centrosome deformation dynamics and fracturing frequency during migration along unidirectional paths, in microenvironmental confinement, and in confining mazes. **(A)** Representative CETN2-GFP (centriole pair; black; enlargement in red dashed boxes) expressing dendritic cells (DCs) stained with Hoechst (nucleus; blue) migrating along a unidirectional straight path (linear microchannel) in the presence of 5 μM GSK-626616 or DMSO (control). See Figures 3D and 3E for quantification. **(B)** CETN2-GFP expressing DCs stained with Hoechst migrating in microenvironmental confinement ('under-agarose assay'), quantifying the distances between individual centrioles in the centriole pair. **(C)** As in (B), classifying the short- and long-distantly separated individual centrioles. **(D)** CETN2-GFP expressing DCs stained with Hoechst migrating in confining and maze-like microenvironments ('under-agarose assay with bead-obstacles'). **(E)** As in (D), quantifying the distances between individual centrioles in the centriole pair. All data derive from at least three independent biological replicates. Time is indicated as h:min:s.

Fig. S5. Development of an 'under-agarose assay with bead obstacles and measurement of centriole distances after centrosome fracturing over time.

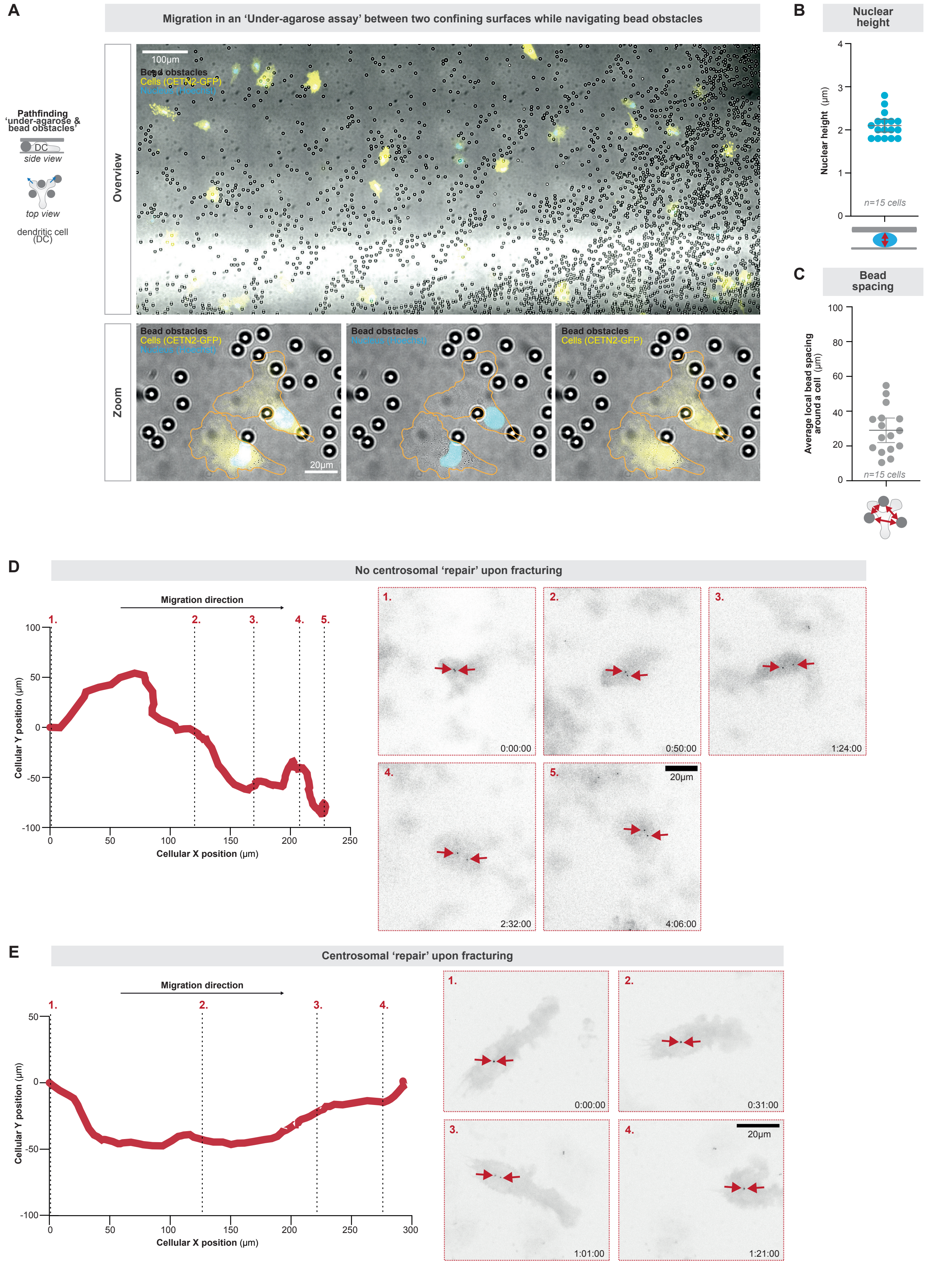

Fig. S5.

Development of an ‘under-agarose assay with bead obstacles’ and measurement of centriole distances after centrosome fracturing over time. **(A)** Representative overview and zoom-in images of an ‘under-agarose assay with bead obstacles’, in which cells migrate between two confining surfaces while navigating bead obstacles. The orange line in the zoom-in images visualizes the cell shape. The CETN2-GFP fluorescence is shown in an overexposed manner to show the shape of the entire cell by showing the CETN2-GFP background signal (see the main figures for normal signal intensities to visualize the pair of centrioles). **(B)** Measurement of the nuclear height by 0.2-micron spaced z-stacks, indicating the available space and confinement degree in the ‘under-agarose assay with bead obstacles’. **(C)** Measurement of the local bead spacing surrounding individual cells, as also visually depicted in (A). **(D)** Representative ‘non-repairing’ CETN2-GFP (centriole pair; black) expressing dendritic cell (DC) migrating in confining and maze-like microenvironments (‘under-agarose assay with bead-obstacles’) in the presence of 5 μM GSK-626616. See Figure 3J for quantification. **(E)** Representative ‘repairing’ CETN2-GFP (centriole pair; black) expressing DC migrating in confining and maze-like microenvironments in the presence of 5 μM GSK-626616. See Figure 3J for quantification. All data show representative cells from at least three independent biological replicates. Time is indicated as h:min:s.

Fig. S6. Sequencing of differentiated dendritic cells with conditional Cas9 expression targeting cNAP1.

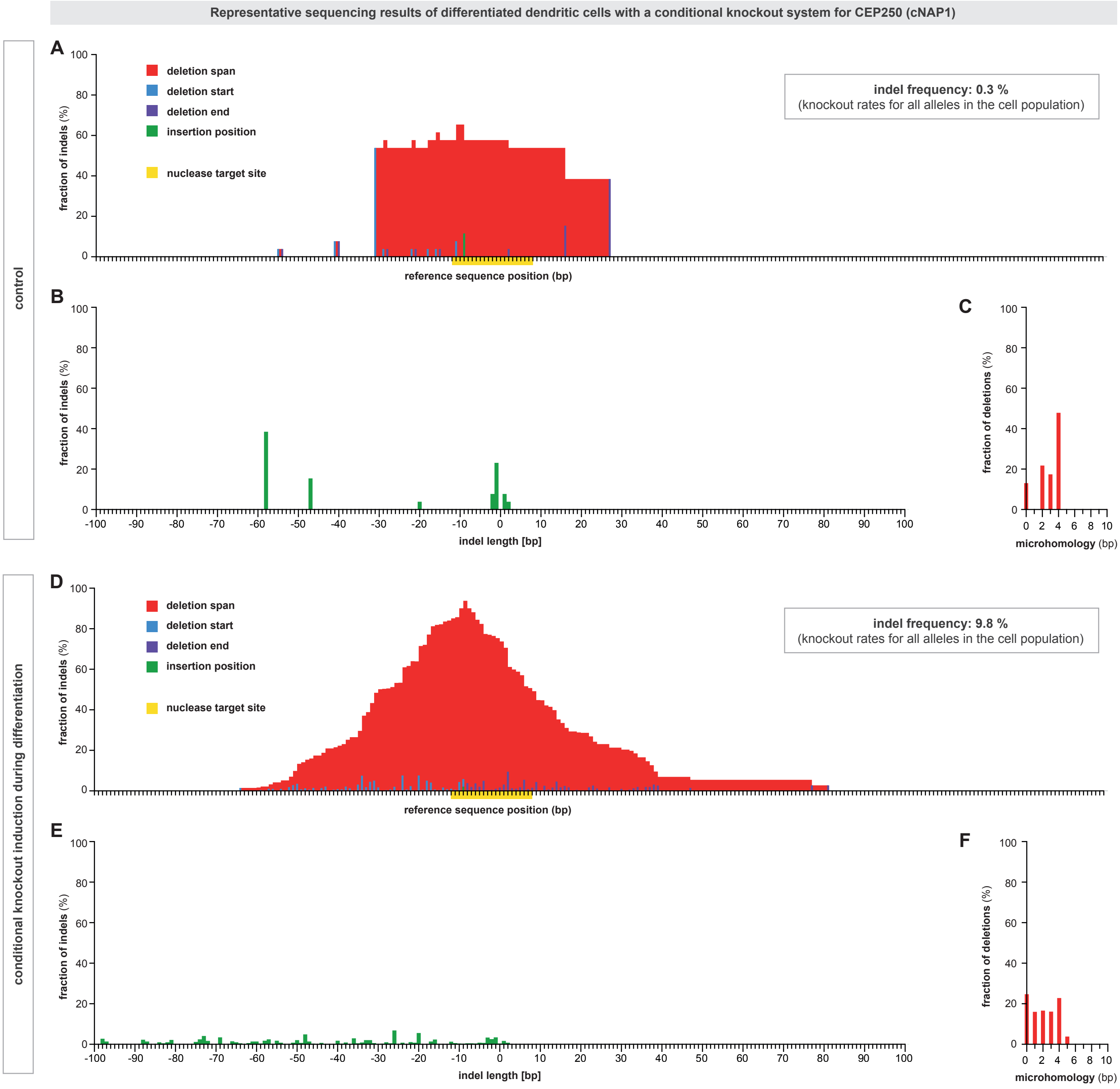

Fig. S6.

Sequencing of differentiated dendritic cells with conditional Cas9 expression targeting cNAP1. **(A)** Representative sequencing result of differentiated dendritic cells (DCs) without induction of Cas9 expression (control) displaying deletion span (red), deletion start (blue), deletion end (purple), insertion position (green) and the Cas9 nuclease target site (yellow). **(B)** Representative sequencing result of differentiated DCs without induction of Cas9 expression (control) displaying indel lengths (green). **(C)** Representative quantification of microhomologies at deletion sites of differentiated DCs without induction of Cas9 expression (control). **(D)** As in (A), but for differentiated DCs with induction of Cas9 expression. **(E)** As in (B), but for differentiated DCs with induction of Cas9 expression. **(F)** As in (C), but for differentiated DCs with induction of Cas9 expression.

Fig. S7. Centrosome deformation during cellular squeezing.

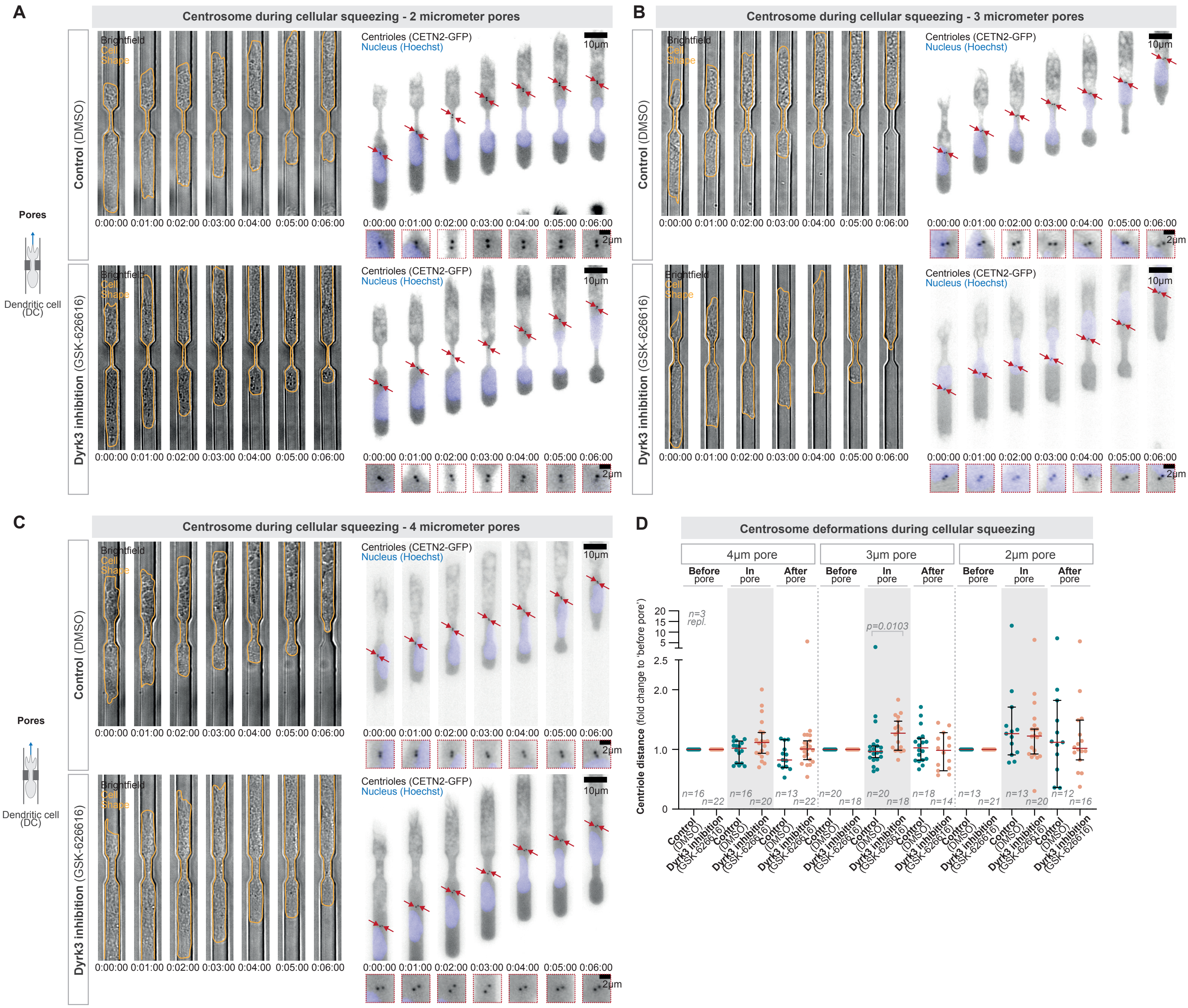

Fig. S7.

Centrosome deformation during cellular squeezing. **(A)** Representative CETN2-GFP (centriole pair; black; enlargement in red dashed boxes) expressing dendritic cells (DCs) stained with Hoechst (nucleus; blue) migrating along a single 2 μm pore (in a linear microchannel) in the presence of 5 μM GSK-626616 or DMSO (control). **(B)** As in (A), but translocation through a 3 μm pore. **(C)** As in (A), but translocation through a 4 μm pore. **(D)** Quantification of centriolar distances in (A) to (C). All data show representative cells from at least three independent biological replicates. Time is indicated as h:min:s.

Fig. S8. Actin forces fracture the centrosome.

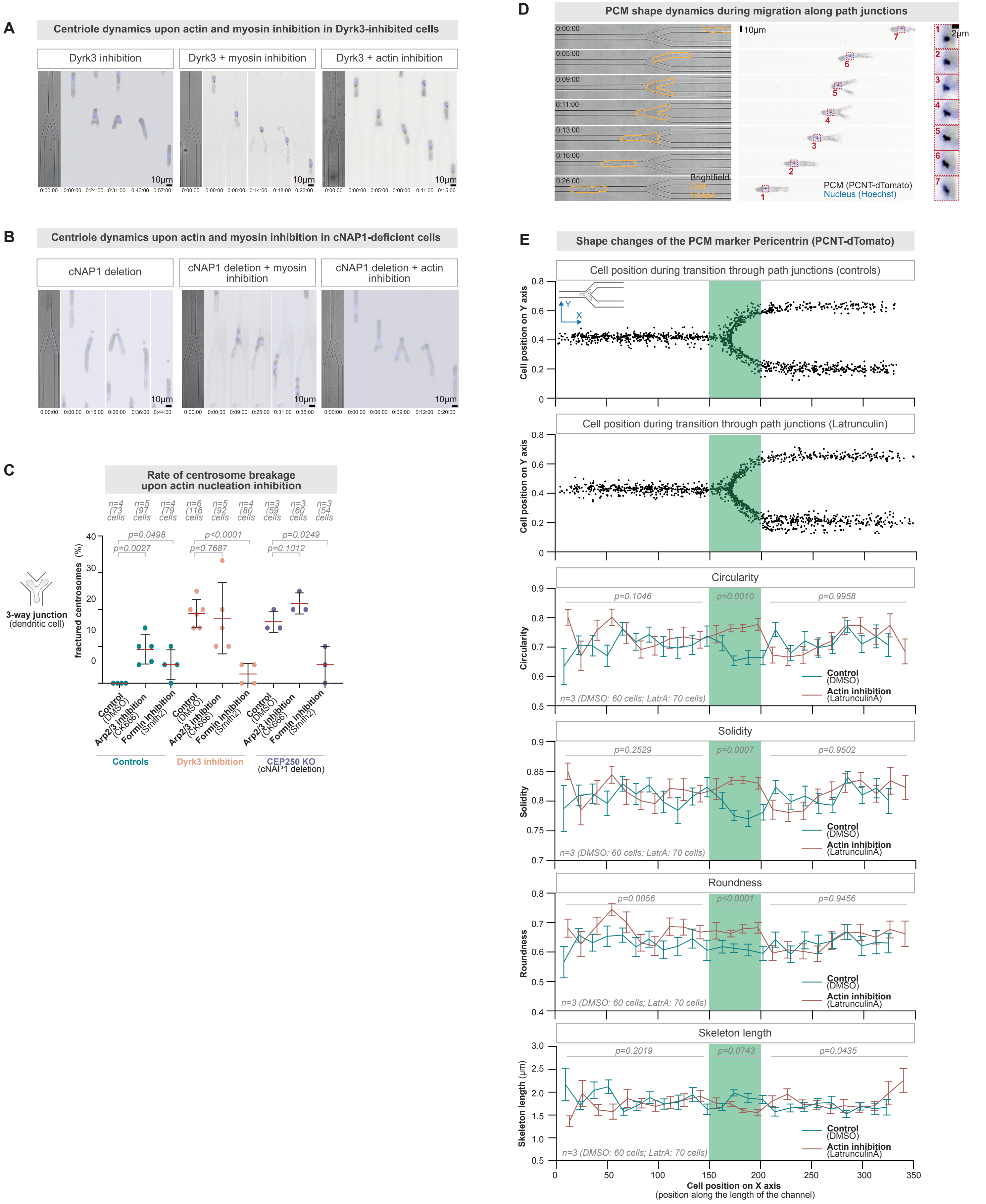

Fig. S8.

Actin forces fracture the centrosome. **(A)** Representative CETN2-GFP (centriole pair; black) expressing dendritic cells (DCs) stained with Hoechst (nucleus; blue) migrating along a 3-way path junction in the presence of 5 μM GSK-626616 and 50 nM Latrunculin A or 5 μM GSK-626616 and 25 μM para-nitro-Blebbistatin. **(B)** Representative CETN2-GFP (centriole pair; black) expressing cNAP1-deficient DCs stained with Hoechst (nucleus; blue) migrating along a 3-way path junction in the presence of 50 nM Latrunculin A or 25 μM para-nitro-Blebbistatin. **(C)** Frequency of centrosome fracturing during DC migration along a 3-way path junction in the presence of 5 μM GSK-626616 or control (DMSO), or upon cNAP1 deletion, and in co-presence of 50 μM CK666 to inhibit Arp2/3-mediated actin nucleation, 15 μM Smifh2 to inhibit formin-based actin nucleation, or control (DMSO). **(D)** Representative PCNT-dTomato (black) expressing DCs stained with Hoechst (nucleus; blue) migrating along a 3-way path. **(E)** Quantification of PCM shape changes in PCNT-dTomato expressing DCs upon migration along 3-way path junctions in the presence of 50 nM Latrunculin A or DMSO (control). All data show representative cells from at least three independent biological replicates. Time is indicated as h:min:s.

Fig. S9. The microtubule cytoskeleton upon Dyrk3 inhibition and centrosome fracturing.

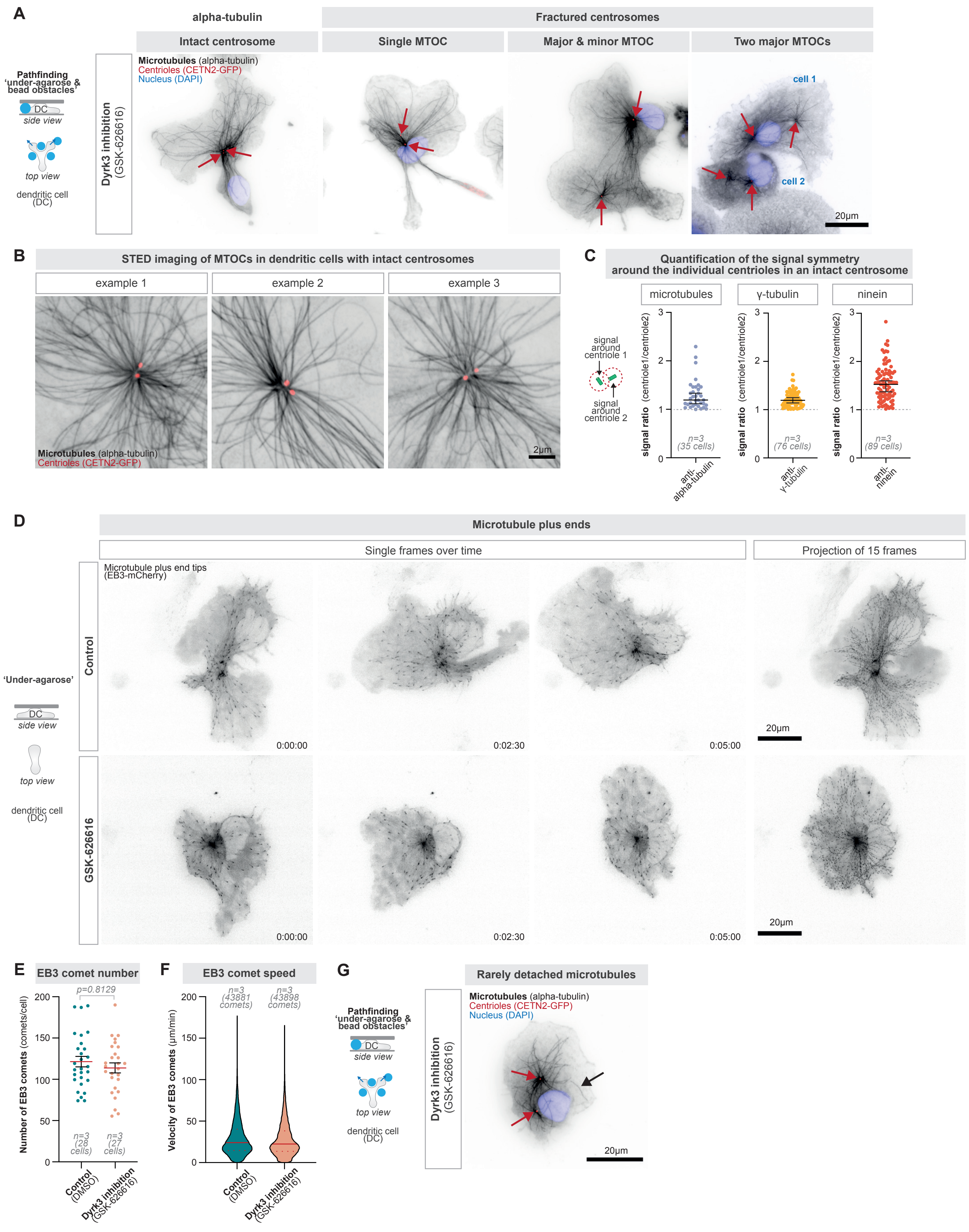

Fig. S9.

The microtubule cytoskeleton upon Dyrk3 inhibition and centrosome fracturing. **(A)** Immunofluorescence staining of representative CETN2-GFP (centriole pair; red) expressing dendritic cells (DCs) stained with DAPI (nucleus; blue) and with alpha-tubulin (black) in the presence of 5 μM GSK-626616. See Figure 5G for quantification. **(B)** Representative stimulated emission depletion (STED) images of microtubules stained with alpha-tubulin (black) in CETN2-GFP (centriole pair; red) expressing dendritic cells (DCs) with intact centrosomes. **(C)** Quantification of signal symmetry of microtubules, the microtubule nucleator gamma-tubulin, and the microtubule anchoring protein ninein around individual centrioles in intact centrosomes. **(D)** Live-cell imaging examples of representative EB3-mCherry (microtubule-plus end tip marker; black) expressing DCs in the presence of 5 μM GSK-626616 or DMSO (control). Time is indicated as h:min:s. **(E)** Quantification of EB3 comets (microtubule plus ends) as shown in (D). **(F)** Quantification of EB3 comet velocity shown as median (red line) and quartiles (red dashed lines). **(G)** Exemplary DC with a rather rare event of a likely non-anchored microtubule (black arrow); CETN2-GFP (centriole pair; red) expressing DCs stained with DAPI (nucleus; blue) and with anti-alpha-tubulin (black) in the presence of 5 μM GSK-626616. All data show representative cells from at least three independent biological replicates.

Fig. S10. Formation of two MTOCs upon centrosome breakage in motile WT cells and live cell imaging of the emergence of 2 MTOCs upon centrosome fracturing following Dyrk3 inhibition.

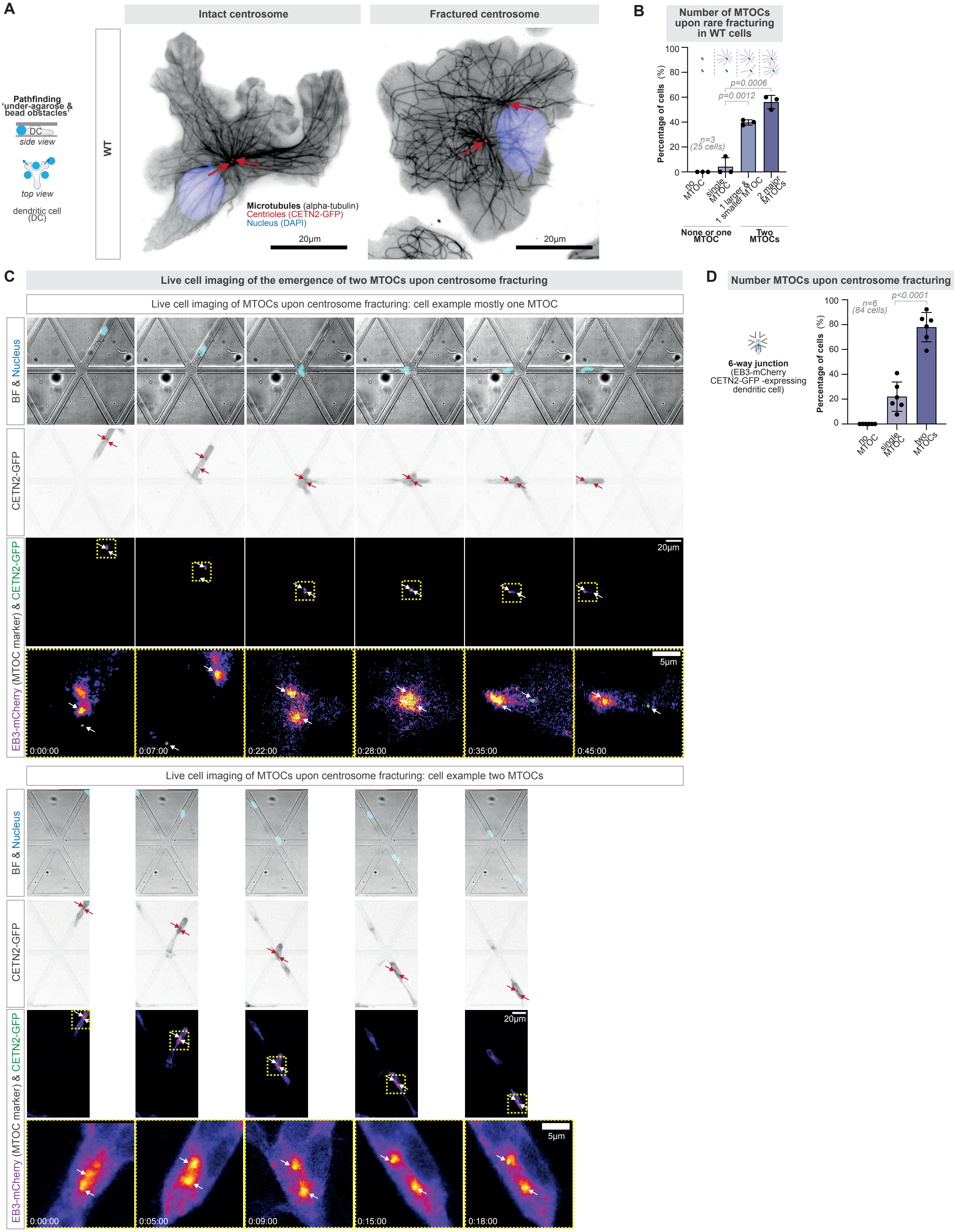

Fig. S10.

Formation of two MTOCs upon centrosome breakage in motile WT cells and live cell imaging of the emergence of two MTOCs upon centrosome fracturing following Dyrk3 inhibition. **(A)** Immunofluorescence staining of representative wildtype CETN2-GFP (red, arrows) expressing dendritic cells (DCs) upon migration through bead-obstacles underneath an agarose layer showing representative examples of intact and fractured centrosomes. Anti-alpha-tubulin (black) and DAPI (blue) visualize the microtubule cytoskeleton and the nucleus, respectively. **(B)** Quantification of microtubule aster formation upon rare breakage of the centrosome in wildtype cells. **(C)** Representative CETN2-GFP (centriole pair; black and green) EB3-mCherry (microtubule plus-end marker; fire-color coded; enlargement in yellow dashed boxes) expressing dendritic cells (DCs) stained with Hoechst (nucleus; cyan) migrating along a 6-way path junction in the presence of 5 μM GSK-626616. **(D)** Quantification of MTOC emergence upon centrosome fracturing as shown in (C). All data show representative cells from at least three independent biological replicates. Time is indicated as h:min:s.

Fig. S11. Microtubule anchoring and nucleating proteins upon Dyrk3 inhibition and upon centrosome fracturing.

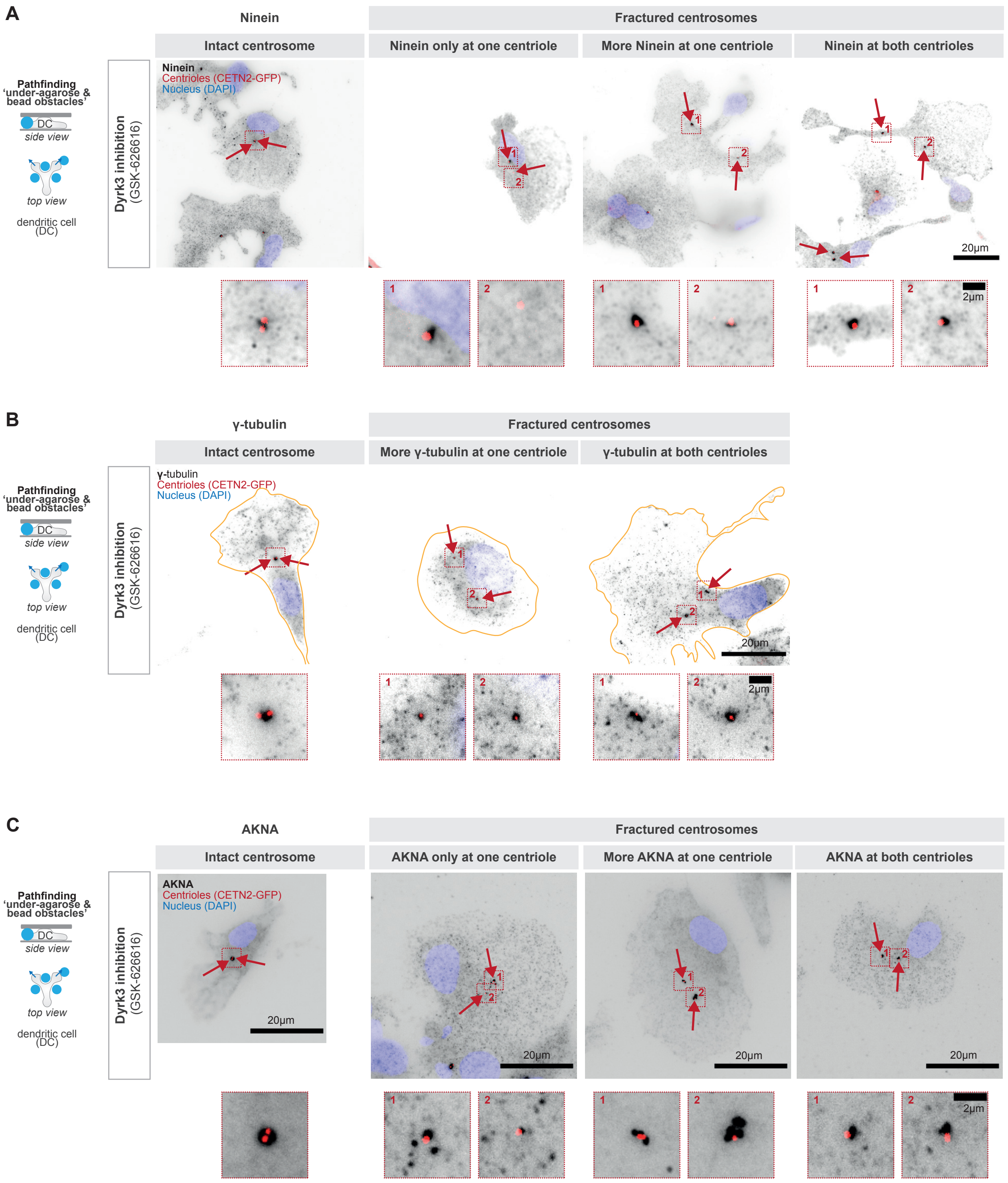

Fig. S11.

Microtubule anchoring and nucleating proteins upon Dyrk3 inhibition and upon centrosome fracturing. **(A)** Immunofluorescence staining of representative CETN2-GFP (centriole pair; red) expressing dendritic cells (DCs) stained with DAPI (nucleus; blue) and with anti-ninein (black) in the presence of 5 μM GSK-626616. See Figure 5H for quantification. **(B)** Immunofluorescence staining of representative CETN2-GFP (centriole pair; red) expressing dendritic cells (DCs) stained with DAPI (nucleus; blue) and with anti-gamma-tubulin (black) in the presence of 5 μM GSK-626616. See Figure 5I for quantification. **(C)** Immunofluorescence staining of representative CETN2-GFP (centriole pair; red) expressing dendritic cells (DCs) stained with DAPI (nucleus; blue) and with anti-AKNA (black) in the presence of 5 μM GSK-626616. See Figure 5J for quantification. All data show representative cells from at least three independent biological replicates.

Fig. S12. Dendritic cell migration phenotypes in the presence of unstable centrosomes (non-functional Dyrk3).

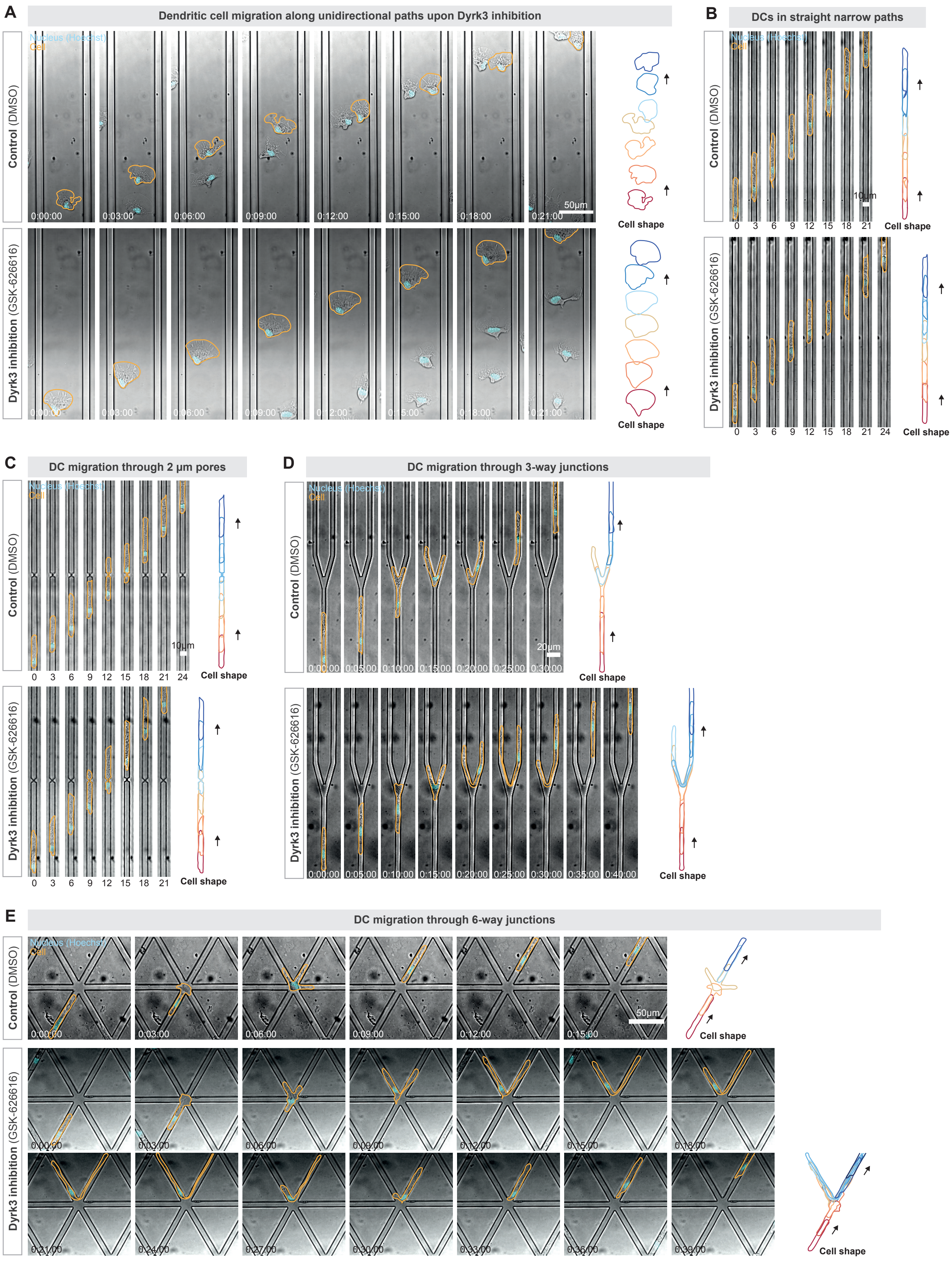

Fig. S12.

Dendritic cell migration phenotypes in the presence of unstable centrosomes (nonfunctional Dyrk3). **(A)** Representative dendritic cells (DCs) migrating along a unidirectional straight path (wide linear microchannel) in the presence of 5 μM GSK-626616 or DMSO (control). See Figure 6A for quantification. **(B)** Representative DCs migrating along a unidirectional straight path (narrow linear microchannel) in the presence of 5 μM GSK-626616 or DMSO (control). See Figure 6A for quantification. **(C)** Representative DCs migrating through a 2 μm pore in the presence of 5 μM GSK-626616 or DMSO (control). See Figure 6B for quantification. **(D)** Representative DCs migrating along a 3-way path junction in the presence of 5 μM GSK-626616 or DMSO (control). See Figure 6C for quantification. **(E)** Representative DCs migrating along a 6-way path junction in the presence of 5 μM GSK-626616 or DMSO (control). See Figure 6C for quantification. All data show representative cells from at least three independent biological replicates. Time is indicated as h:min:s.

Fig. S13. Jurkat T cell migration phenotypes in the presence of unstable centrosomes (non-functional Dyrk3).

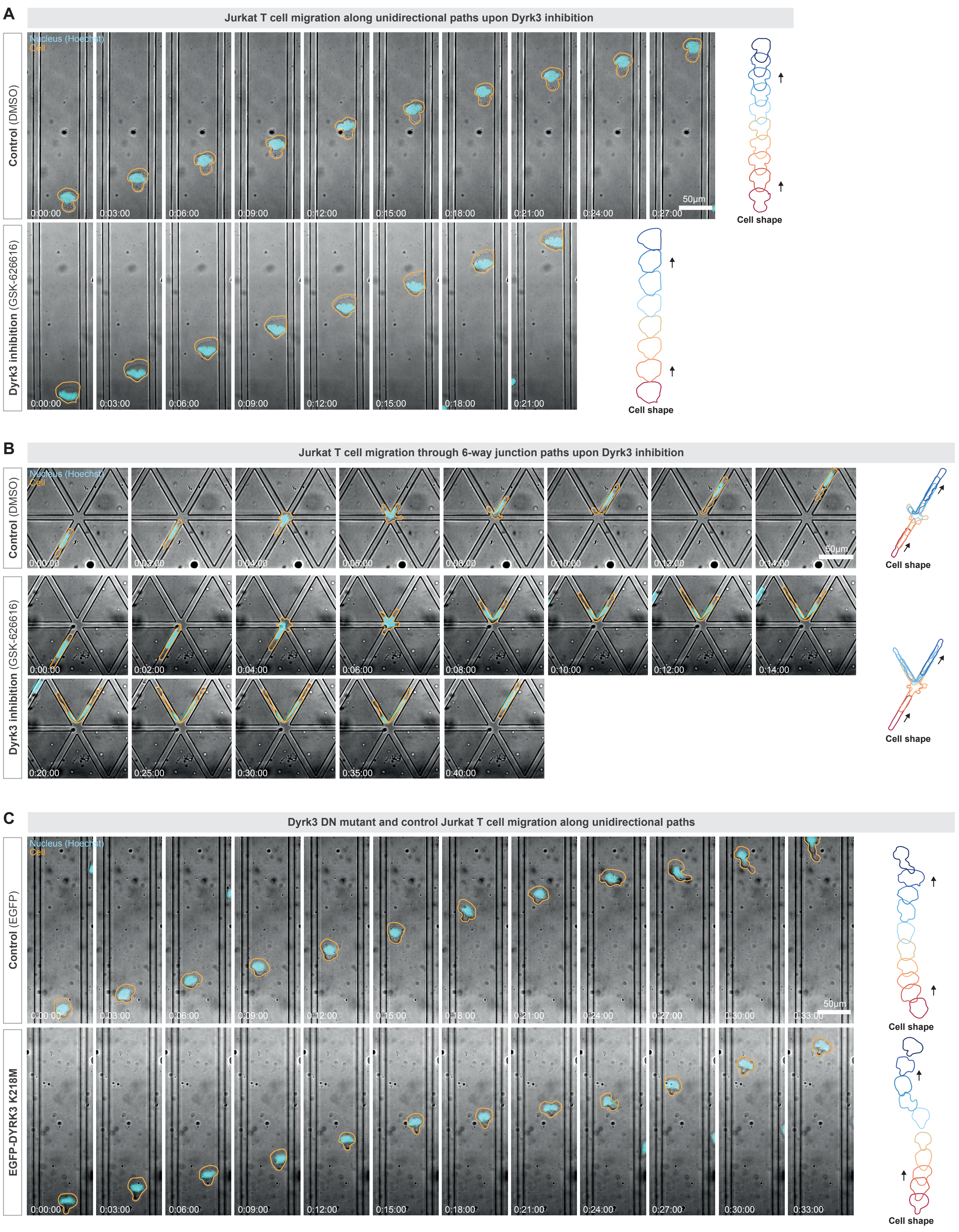

Fig. S13.

Jurkat T cell migration phenotypes in the presence of unstable centrosomes (nonfunctional Dyrk3). **(A)** Representative Jurkat T cell migrating along a unidirectional straight path (wide linear microchannel) in the presence of 5 μM GSK-626616 or DMSO (control). See Figure 6D for quantification. **(B)** Representative Jurkat T cell migrating along a 6-way path junction in the presence of 5 μM GSK-626616 or DMSO (control). See Figure 6D for quantification. **(C)** Representative Jurkat T cell expressing EGFP-Dyrk3 K218M or only EGFP while migrating along a unidirectional straight path (wide linear microchannel). See Figure 6F for quantification, and Figure 6E for comparison to migration through path junctions. All data show representative cells from at least three independent biological replicates. Time is indicated as h:min:s.

Fig. S14. Altered cell shapes during dendritic cell and Jurkat T cell migration in 3D collagen matrices upon rendering Dyrk3 non-functional.

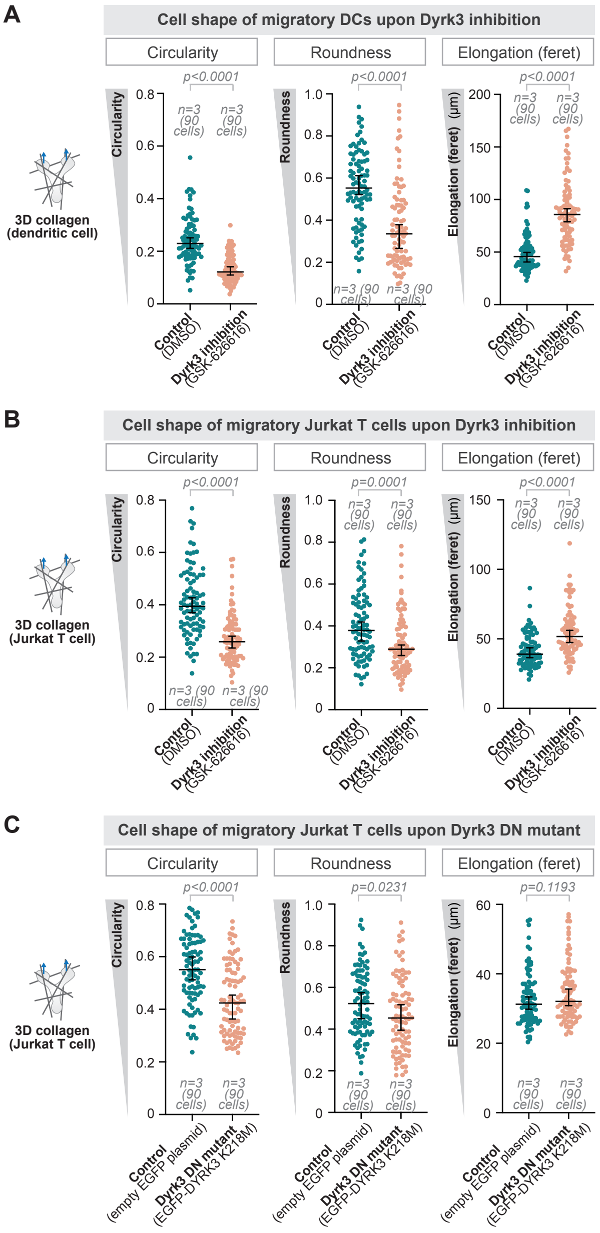

Fig. S14.

Altered cell shapes during dendritic cell and Jurkat T cells migration in 3D collagen matrices upon rendering Dyrk3 non-functional. **(A)** Circularity, roundness, and elongation of dendritic cells migrating in 3D collagen matrices (1.7 mg/ml) along a CCL19 chemokine gradient in the presence of 5 μM GSK-626616 or DMSO (control). **(B)** Circularity, roundness, and elongation of Jurkat T cells migrating in 3D collagen matrices (1.3 mg/ml) along a CXCL12 chemokine gradient in the presence of 5 μM GSK-626616 or DMSO (control). **(C)** Circularity, roundness, and elongation of Jurkat T cells that express a dominant-negative (DN) EGFP-Dyrk3 K218M mutant or the corresponding empty EGFP plasmid migrating in 3D collagen matrices (1.3 mg/ml) along a CXCL12 chemokine gradient. All data derive from at least three independent biological replicates.

Fig. S15. Dendritic cells retain their polarity upon rendering Dyrk3 non-functional.

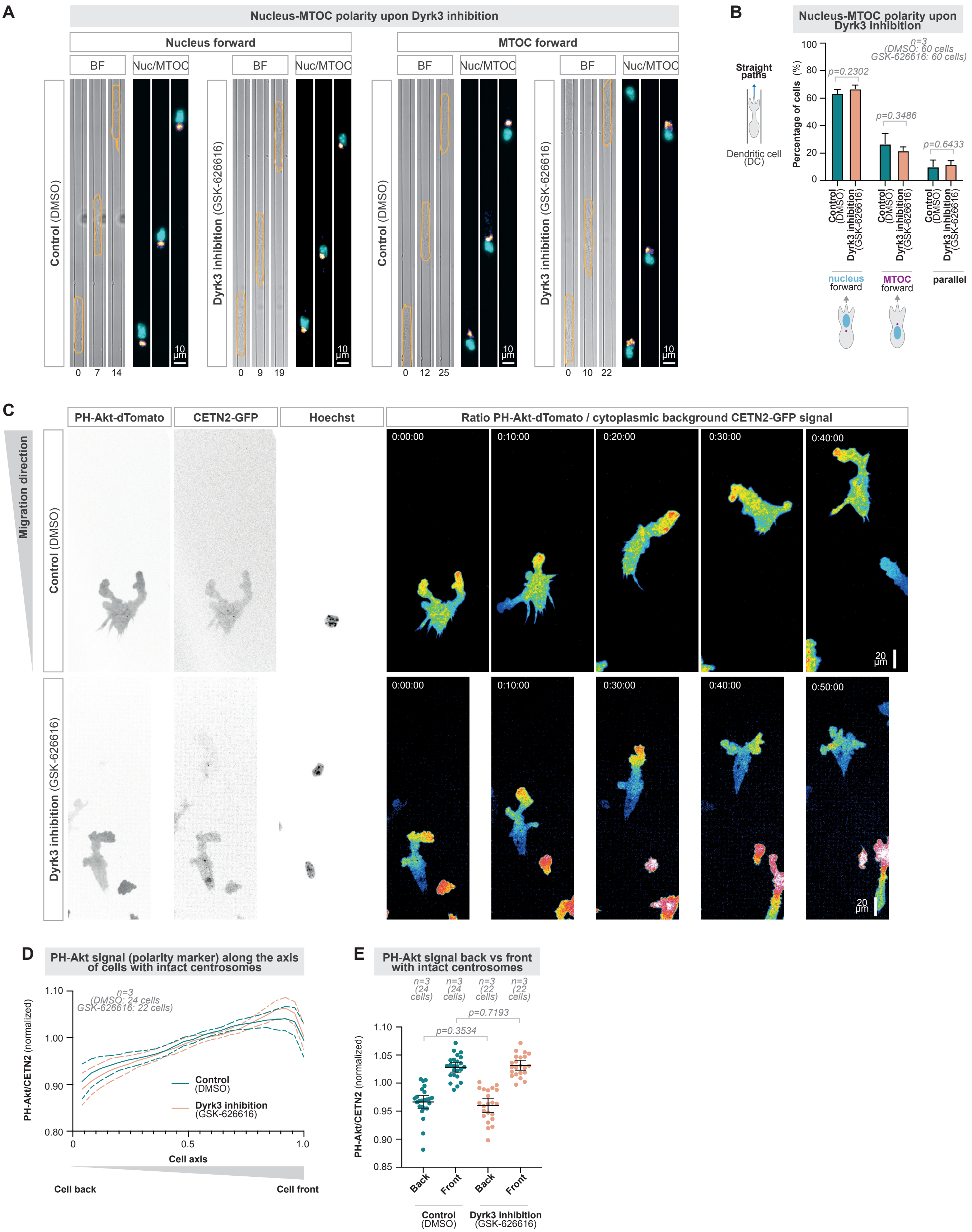

Fig. S15.

Dendritic cells retain their polarity upon rendering Dyrk3 non-functional. **(A)** Representative EMTB-mCherry (microtubule organizing center (MTOC) marker; fire-color coded) expressing dendritic cells (DCs) stained with Hoechst (nucleus; cyan) migrating along a unidirectional straight path (narrow linear microchannel) in the presence of 5 μM GSK-626616 or DMSO (control). Time is indicated in minutes. **(B)** Quantification of nucleus-MTOC axis configuration as shown in (A). **(C)** Representative CETN2-GFP (centriole pair; black) PH-Akt-dTomato (polarity marker; black) expressing DCs stained with Hoechst (nucleus; black) migrating in microenvironmental confinement ('under-agarose assay') in the presence of 5 μM GSK-626616 or DMSO (control). Fluorescence ratio images show the PH-Akt-dTomato signal normalized to the CETN2-GFP signal (see 'Material and Methods' for details). **(D)** Quantification of normalized PH-Akt signal distribution along the cell axis of migrating DCs with intact centrosomes as shown in (C). **(E)** As in (D), quantifying normalized PH-Akt signal in the front and back half of migrating DCs. All data derive from at least three independent biological replicates. Time is indicated as h:min:s.

Fig. S16. Dendritic cell race in the context of broken centrosomes shows impaired cellular navigation upon centrosome fracturing.

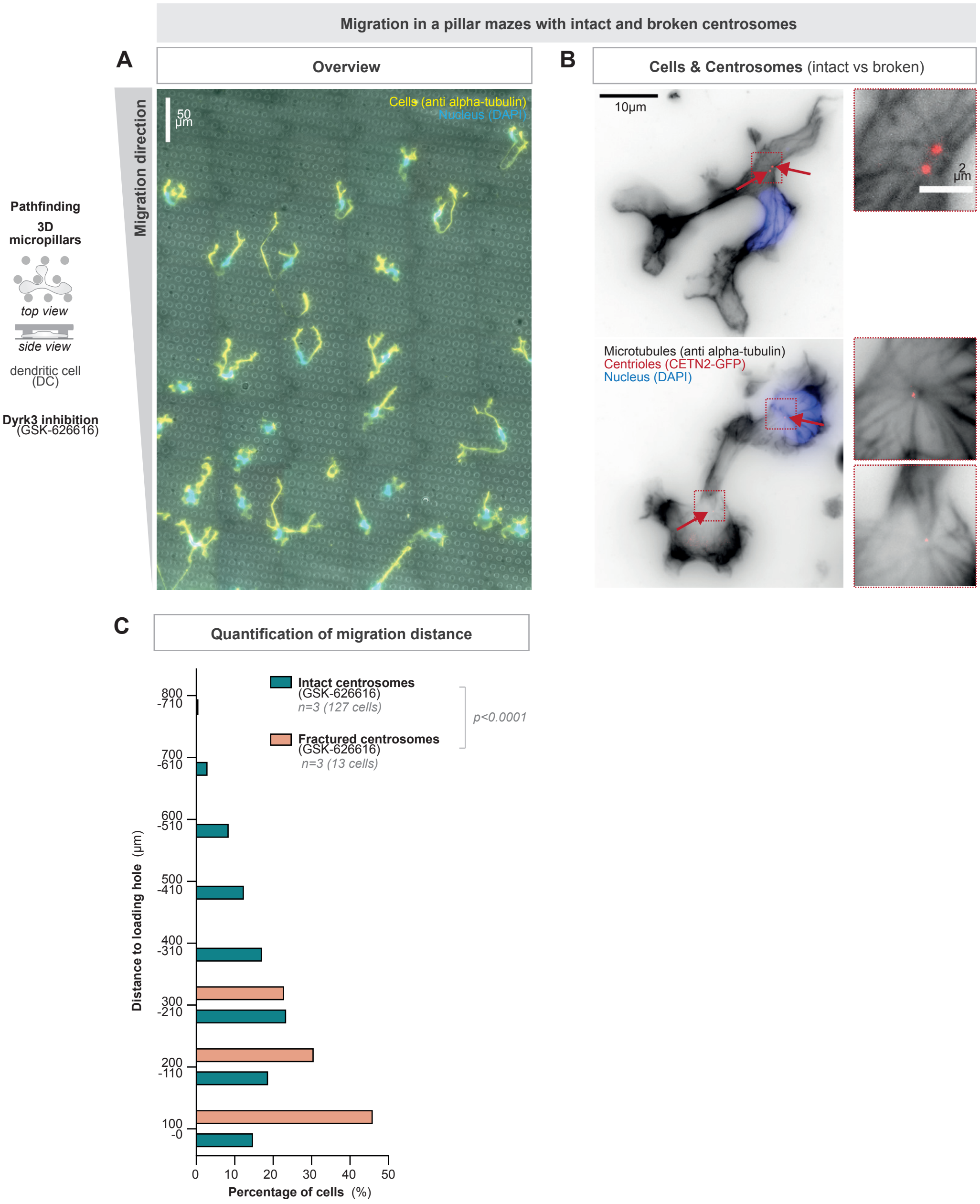

Fig. S16.

Dendritic cell race in the context of broken centrosomes shows impaired cellular navigation upon centrosome fracturing. **(A)** Representative overview of a 'cell race', in which cells migrate towards a CCL19 source while navigating through 3D micropillars in the presence of 5 μM GSK-626616 (Dyrk3 inhibitor). The alpha-tubulin fluorescence is shown in an overexposed manner to show the shape of the entire cell (see (B) for normal signal intensities to visualize the microtubule network). **(B)** Immunofluorescence staining of CETN2-GFP (red, arrows) expressing dendritic cells (DCs) upon migration through 3D micropillars in the presence of 5 μM GSK-626616 (Dyrk3 inhibitor), showing representative examples of cells with an intact and fractured centrosome. Anti-alpha-tubulin (black) and DAPI (blue) visualize the microtubule cytoskeleton and the nucleus, respectively. **(C)** Migrated distance of CETN2-GFP expressing DCs with intact or fractured centrosomes migrating through 3D micropillars in the presence of 5 μM GSK-626616.

Fig. S17. Dendritic cell migration without centrioles.

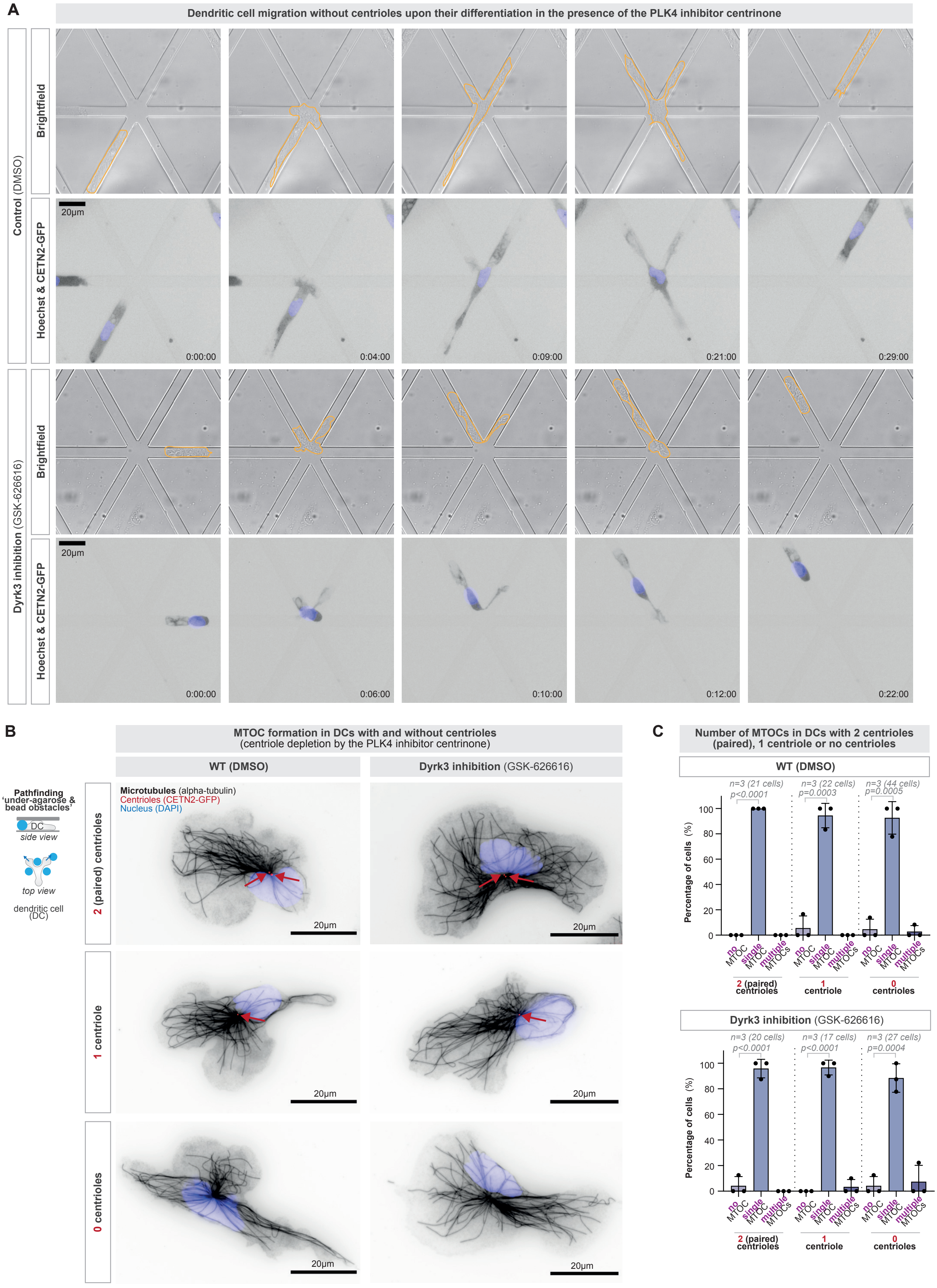

Fig. S17.

Dendritic cell migration without centrioles. **(A)** Representative centriole-depleted CETN2-GFP (absent centriole pair; black) expressing dendritic cells (DCs) stained with Hoechst (nucleus; blue) migrating along a 6-way path junction in the presence of 5 μM GSK-626616 or DMSO (control). **(B)** Immunofluorescence staining of PLK4-inhibited (500 nM centrinone) CETN2-GFP (red, arrows) expressing dendritic cells (DCs) upon migration through bead-obstacles underneath an agarose layer in the presence of 5 μM GSK-626616 or control (DMSO), showing representative examples of two paired, one or no centrioles. Anti-alpha-tubulin (black) and DAPI (blue) visualize the microtubule cytoskeleton and the nucleus, respectively. **(C)** Quantification of MTOC formation in DCs with and without centrioles in the presence of 5 μM GSK-626616 or control (DMSO). All data show representative cells from at least three independent biological replicates. Time is indicated as h:min:s.

Movie S1.

Centrosome deformations during cellular navigation. Representative CETN2-GFP expressing dendritic cells migrating along path junctions (1st movie part) and unidirectional paths (2nd movie part) in chemokine gradient (CCL19). The pair of centrioles (CETN2-GFP) is shown in black and the nucleus (Hoechst) is shown in blue. Note the short-range transient separation of the centriole pair (red arrow) in particular during cellular path decisions at path junctions. The movie shows representative cells from at least three independent biological replicates. Time is indicated as h:min:s.

Movie S2.

Rendering Dyrk3 non-functional impairs cell migration in three-dimensional collagen matrices. Representative dendritic cells (1st movie part) and Jurkat T cells (2nd and 3rd movie part) migrating in three-dimensional collagen matrices in a chemokine gradient (CCL19), either in the presence of the Dyrk3 inhibitor GSK-626616 (5 μM) or DMSO control (1st and 2nd movie part) or in the presence of Dyrk3-K218M-GFP (3rd movie part). The movie shows representative cells from at least three independent biological replicates. Time is indicated as h:min:s.

Movie S3.

Centrosome fracturing during cellular navigation upon rendering Dyrk3 non-functional. Representative CETN2-GFP dendritic cells migrating along path junctions (1st movie part) and unidirectional paths (2nd movie part) in the presence of the Dyrk3 inhibitor GSK-626616 (5 μM) or DMSO control. The pair of centrioles (CETN2-GFP) is shown in black and the nucleus (Hoechst) is shown in blue. Note the long-range separation of the centriole pair (red arrow) during cellular path decisions in the presence of the Dyrk3 inhibitor GSK-626616. The movie shows representative cells from at least three independent biological replicates. Time is indicated as h:min:s.

Movie S4.

Centrosome deformations during cellular squeezing. Representative CETN2-GFP dendritic cells migrating through 2 micrometer pores (1st movie part) and 3 micrometer pores (2nd movie part) in the presence of the Dyrk3 inhibitor GSK-626616 (5 μM) or DMSO control. The pair of centrioles (CETN2-GFP) is shown in black; the nucleus (Hoechst) is shown in blue. Note the short-range centrosome deformations during cellular squeezing, which already occurs during translocation through 3 micrometer pores in the presence of the Dyrk3 inhibitor GSK-626616 (5 μM). The movie shows representative cells from at least three independent biological replicates. Time is indicated as h:min:s.

Movie S5.

Centrosome fracturing is mediated by forces from the actin cytoskeleton. Representative CETN2-GFP expressing dendritic cells migrating along a 3-way path junction in the presence of GSK-626616 (5 μM; left) alone, or in co-presence of the myosin inhibitor para-nitro-Blebbistatin (25 μM; middle) or the actin inhibitor Latrunculin A (50 nM; right). The pair of centrioles (CETN2-GFP; red arrows) is shown in black, and the nucleus (Hoechst) is shown in blue. The movie shows representative cells from at least three independent biological replicates. Time is indicated as h:min.

Movie S6.

Microtubule dynamics in the presence of the Dyrk3 inhibitor GSK-626616. Representative EB3-mCherry (microtubule plus-end binding) expressing dendritic cells in a confining "under-agarose" environment in the presence of the Dyrk3 inhibitor GSK-626616 (5 μM) or DMSO control. Growing microtubule plus end tips (EB3-mCherry) are shown in black. The movie shows representative cells from at least three independent biological replicates. Time is indicated as h:min:s.

Movie S7.

Dendritic cell migration through path junctions and unidirectional paths in the presence of unstable centrosomes (non-functional Dyrk3). Representative dendritic cells migrating through wide linear paths (1st movie part), narrow linear paths (2nd movie part), 2 micrometer pores (3rd movie part), 3-way junctions (4th movie part), and 6-way junctions (5th movie part) in the presence of the Dyrk3 inhibitor GSK-626616 (5 μM) or DMSO control. The nucleus (Hoechst) is shown in cyan. The movie shows representative cells from at least three independent biological replicates. Time is indicated as h:min:s.

Movie S8.

Jurkat T cell migration through path junctions and unidirectional paths in the presence of unstable centrosomes (non-functional Dyrk3). Representative Jurkat T cells migrating through wide linear paths (1st movie part) or 6-way junctions (2nd movie part) in the presence of the Dyrk3 inhibitor GSK-626616 (5 μM) or DMSO control, and representative Jurkat T cells expressing either EGFP (control) or a Dyrk3 mutant (EGFP Dyrk3 K218M) that are migrating through wide linear paths (3rd movie part) or 6-way junctions (4th movie part). The nucleus (Hoechst) is shown in cyan. The movie shows representative cells from at least three independent biological replicates. Time is indicated as h:min:s.

Movie S9.

Cell polarity during cellular navigation upon rendering Dyrk3 non-functional. Representative EMTB-mCherry expressing dendritic cells migrating along narrow linear paths in a nucleus-forward (1st movie part) and MTOC-forward (2nd movie part) cell axis configuration in the presence of the Dyrk3 inhibitor GSK-626616 (5 μM) or DMSO control. The nucleus (Hoechst) is shown in cyan and the MTOC (EB3-mCherry) is shown in fire-color coding. Representative PH-Akt-dTomato CETN2-GFP expressing dendritic cells shown as fluorescence ratio of the PH-Akt-dTomato signal normalized to the CETN2-GFP signal in a confining "under-agarose" environment in the presence of the Dyrk3 inhibitor GSK-626616 (5 μM) or DMSO control (3rd movie part). The movie shows representative cells from at least three independent biological replicates. Time is indicated as h:min.

Movie S10.

Dendritic cell migration without centrioles through path junctions. Representative CETN2-GFP expressing dendritic cells without centrioles migrating along a 6-way path junction in the presence of GSK-626616 (5 μM) or DMSO (control). CETN2-GFP (note the absent centriole pair) is shown in black, and the nucleus (Hoechst) is shown in blue. The movie shows representative cells from at least three independent biological replicates. Time is indicated as h:min.
